# Aberrant axon initial segment plasticity and intrinsic excitability of ALS hiPSC motor neurons

**DOI:** 10.1016/j.celrep.2023.113509

**Published:** 2023-11-28

**Authors:** Peter Harley, Caoimhe Kerins, Ariana Gatt, Guilherme Neves, Federica Riccio, Carolina Barcellos Machado, Aimee Cheesbrough, Lea R’Bibo, Juan Burrone, Ivo Lieberam

**Affiliations:** 1 Centre for Gene Therapy & Regenerative Medicine, Kings College London, London SE1 9RT, UK; 2 Centre for Developmental Neurobiology, Kings College London, London SE1 1UL, UK; 3 UCL Queen Square Department of Neuromuscular Diseases, UCL Queen Square Institute of Neurology, UCL, London, UK; 4 Centre for Craniofacial & Regenerative Biology, King’s College London, London SE1 9RT, UK; 5 Queen Square Brain Bank, Department of Neurodegenerative Disease, Institute of Neurology, University College London, London WC1N 1PJ, UK; 6 MRC Centre for Neurodevelopmental Disorders, Kings College London, London SE1 1UL, UK

## Abstract

Dysregulated neuronal excitability is a hallmark of amyotrophic lateral sclerosis (ALS). We sought to investigate how functional changes to the axon initial segment (AIS), the site of action potential generation, could impact neuronal excitability in ALS human induced pluripotent stem cell (hiPSC) motor neurons. We find that early *TDP-43* and *C9orf72* hiPSC motor neurons show an increase in the length of the AIS and impaired activity-dependent AIS plasticity that is linked to abnormal homeostatic regulation of neuronal activity and intrinsic hyperexcitability. In turn, these hyperactive neurons drive increased spontaneous myofiber contractions of *in vitro* hiPSC motor units. In contrast, late hiPSC and postmortem ALS motor neurons show AIS shortening, and hiPSC motor neurons progress to hypoexcitability. At a molecular level, aberrant expression of the AIS master scaffolding protein ankyrin-G and AIS-specific voltage-gated sodium channels mirror these dynamic changes in AIS function and excitability. Our results point toward the AIS as an important site of dysfunction in ALS motor neurons.

## Graphical abstract

**Graphical abstract:**
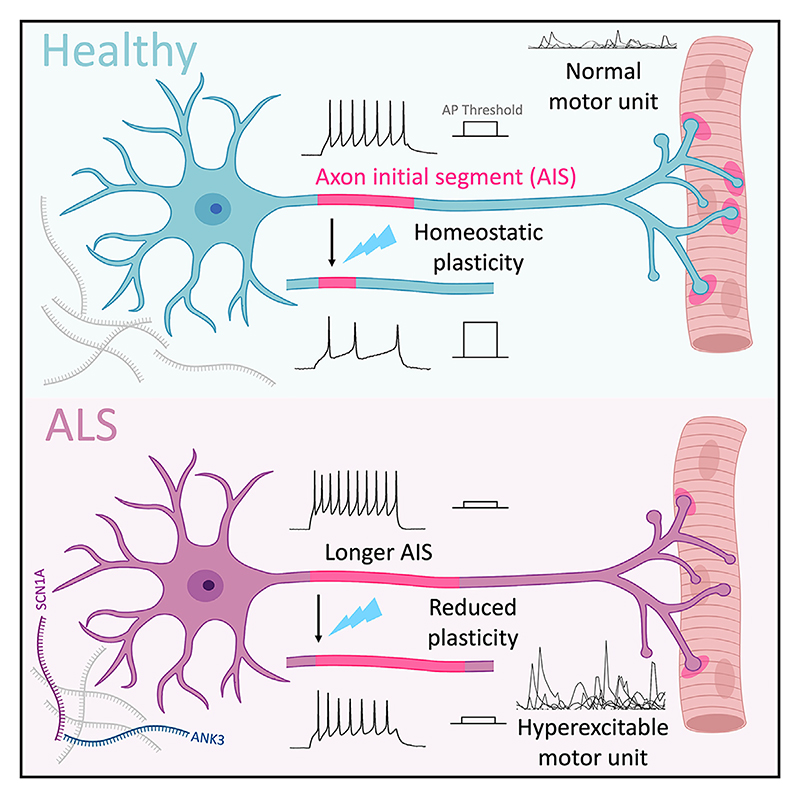

## Introduction

Amyotrophic lateral sclerosis (ALS) is a fatal neuromuscular disease characterized by progressive degeneration of motor neurons (MNs) in the brain and spinal cord.^1^ In the majority of patients, including those with TDP-43 (*TARDBP*) mutations and *C9orf72* hexanucleotide expansions, TDP-43 protein mis-localizes to the cytoplasm and forms ubiquitinated and hyperphosphorylated aggregates,^2–5^ which has been linked to abnormal RNA metabolism and stability, as well as changes to RNA splicing and gene expression.^1,6,7^

Dysregulated neuronal activity, characterized by early hyper-excitability and subsequent progression to hypoexcitability, is a key pathological hallmark of ALS and one of several important diagnostic criteria for the disease.^8,9^ Peripheral axonal and motor unit hyperexcitability caused by abnormal Na+ conductances drives muscle fasciculations (spontaneous contractions) in the early stages of the disease,^10–12^ which has been shown to correlate with increased disease severity and reduced survival time.^13^ Furthermore, riluzole, one of the few approved drugs to extend lifespan in ALS, primarily acts to dampen neuronal excitability by inhibiting tetrodotoxin (TTX)-sensitive sodium channels, showing that modulation of neuronal excitability can modify disease outcomes in patients.^14–16^

The axon initial segment (AIS) is a specialized region of the proximal axon where action potentials are initiated.^17^ This subcellular domain is characterized by a high density of voltage-gated sodium and potassium channels, particularly Nav1.1 (*SCN1A*), Nav1.2 (*SCN2A*), Nav1.6 (*SCN8A*), Kv1.1 (*KCNA1*), and Kv7.2/3 (*KCNQ2/3*), that are anchored to the membrane by a unique cytoskeletal arrangement of scaffolding proteins including the master organizer ankyrin-G (*ANK3*).^18,19^ Experimental and computational approaches have shown that AIS length is a crucial determinant of sodium conductance during action potential (AP) initiation and enables neuronal excitability and output to be precisely regulated at a specific site, downstream of synaptic and somato-dendritic influences.^17,20–22^

Indeed, key experimental studies have shown that the AIS can undergo unique forms of activity-dependent plasticity, whereby elevated activity leads to either a shortening or distal relocation of the AIS, while reduced activity can result in the opposite phenotype.^22–27^ This has been proposed as a form of homeostatic plasticity important for regulating intrinsic excitability, stabilizing neuronal output, and preventing abnormal levels of network activity.

In this study, we sought to determine whether structural, functional, and molecular changes to the AIS could drive abnormal regulation of intrinsic neuronal excitability in ALS MNs and motor units.

## Results

### Increased AIS length and intrinsic hyperexcitability of early ALS patient-derived MNs

**Figure 1:**
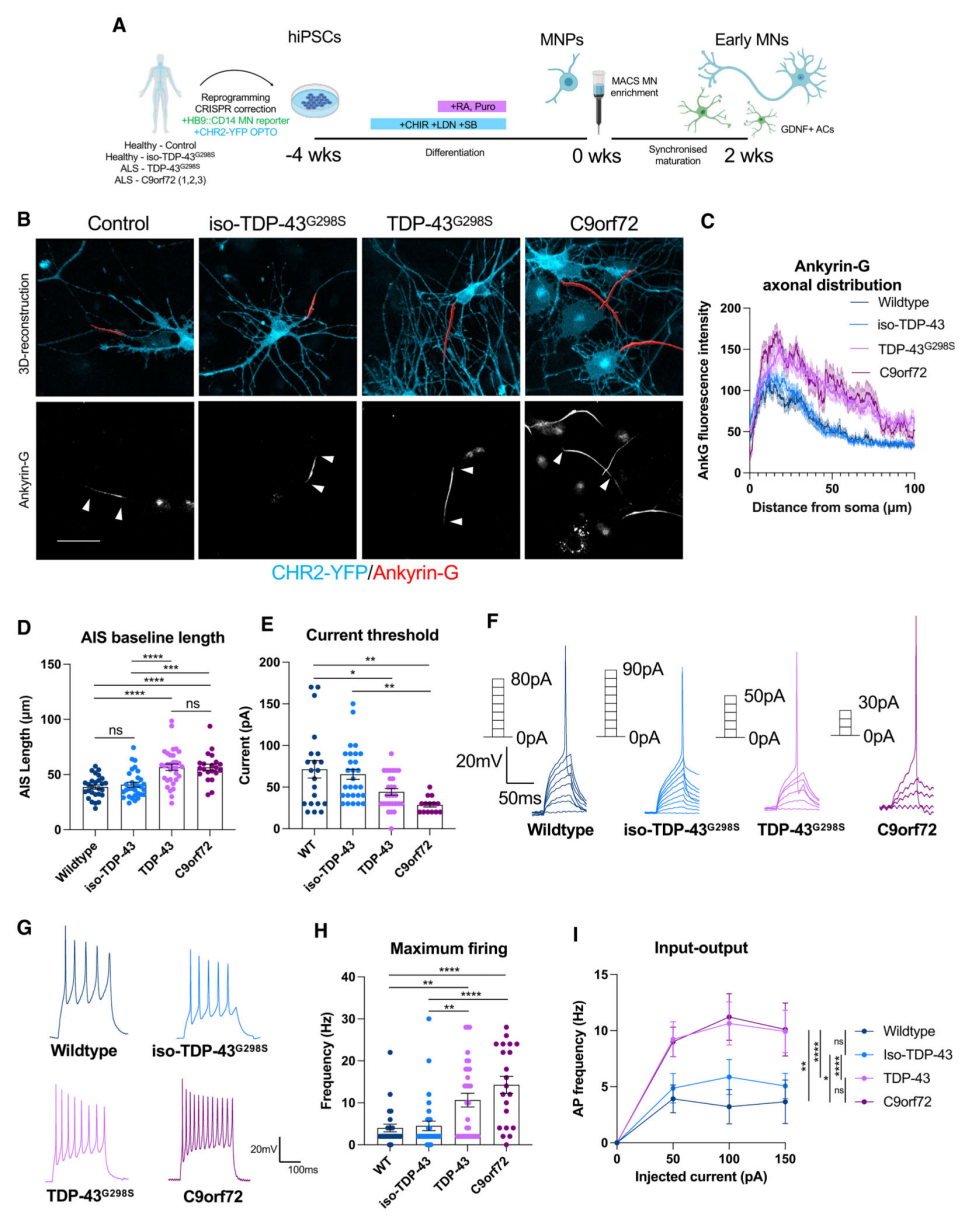
Increased AIS length and intrinsic hyperexcitability of early (2 week) ALS patient-derived motor neurons (A) Schematic showing differentiation of hiPSCs into early MNs. (B) Reconstructions of the AIS (red) pooled from 3 independent experiments in 2 week wild-type (n = 27), iso-TDP-43^G298S^ (n = 31), TDP-43^G298S^ (n = 32), and C9orf72-3 (n = 21) MNs based on AnkG immunofluorescence staining. Counterstained against CHR2-YFP (cyan). White arrows indicate start and end positions. Scale bar: 50 μm. (C) Intensity profile showing distribution of AnkG protein along the axon. (D) Quantification of AIS length in baseline conditions. (E) Whole-cell patch-clamp recordings pooled from 3 independent experiments of current threshold for AP firing in 2 week wild type (n = 21), iso-TDP-43^G298S^ (n = 28), TDP-43^G298S^ (n = 28), and C9orf72-3 (n = 17). (F) Representative single AP traces taken from current clamp recordings. (G) Representative repetitive AP traces taken from current clamp recordings. (H) Maximum evoked AP firing frequency taken from current clamp recordings. (I) Relationship between injected current and firing frequency. Error bars represent the SEM. p values from one-way ANOVA tests with Tukey’s comparison, except 1 h (one-way ANOVA with repeated measures). *p < 0.05, **p < 0.01, ***p < 0.001, and ****p < 0.0001. See also Figures S1 and S2 and Table S1.

*TDP-43*^*G298S*^ and *C9orf72-3* magnetically activated cell-sorted (MACS) HB9+ MNs (Figure S2D) showed an increase in the baseline unstimulated length of the AIS relative to isogenic (Figures S2A–S2C) and wild-type control MNs (Figures 1B–1D). Analysis of two additional, non-engineered C9orf72-1 and C9orf72-2 lines also showed an increase in AIS length relative to CRISPR-corrected isogenic controls (Figure S1A). Consistent with the increase in AIS length, whole-cell patch-clamp recordings revealed that *TDP-43*^*G298S*^ and *C9orf72-3* MNs were hyperexcitable relative to controls, evidenced by a reduction in the current threshold for AP spiking (Figures 1E and 1F) and an increase in the maximum firing frequency (Figures 1G and 1H), as well as a shift in the input-output relationship of injected current to AP firing (Figure 1I). Furthermore, an increase in the inward current (predominantly Na+) was observed, as well as altered AP properties such as increased amplitude and reduced half-width (Figures S4B–S4E), consistent with an increase in AIS length. These changes in excitability could not be explained by changes in passive properties (Table S1).

### Impaired activity-dependent AIS plasticity and homeostatic regulation of intrinsic excitability in early ALS patient-derived MNs

**Figure 2:**
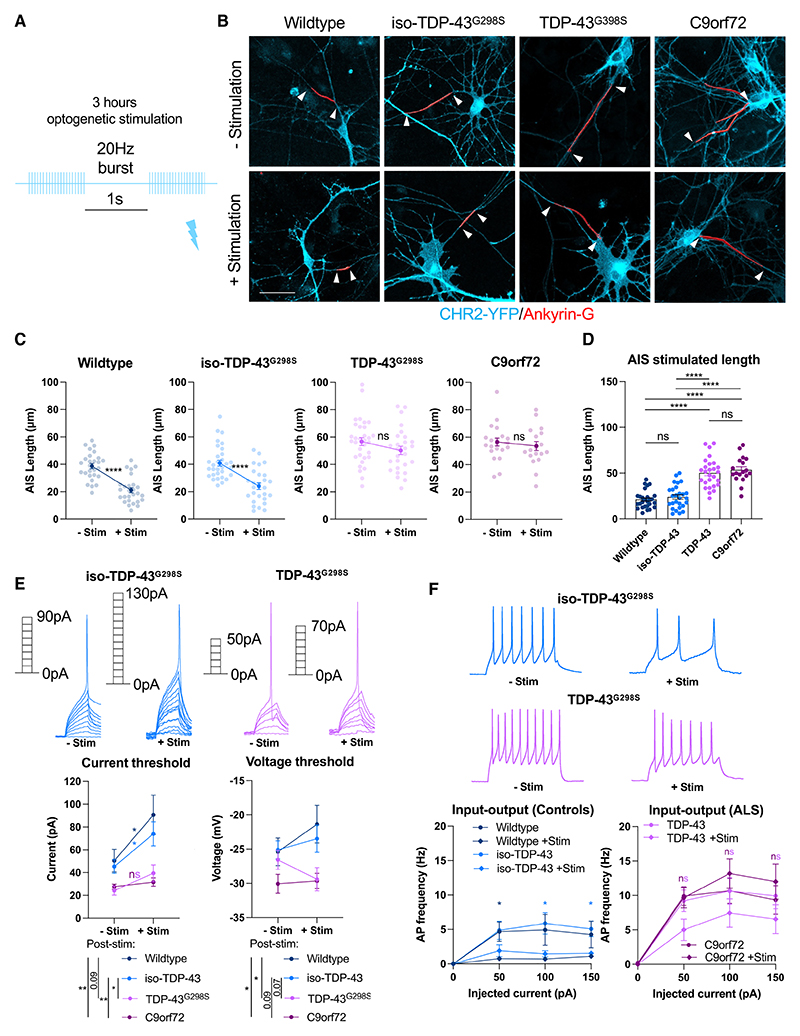
Impaired activity-dependent AIS plasticity and homeostatic regulation of intrinsic excitability in early (2 week) ALS patient-derived motor neurons (A) Schematic showing patterned optogenetic stimulation paradigm for early MNs. (B) Reconstructions of the AIS (red) in 2 week neurons without and with 3 h 5 Hz 488 nm optogenetic stimulation, based on AnkG immunofluorescence staining and counterstaining against CHR2-YFP (cyan). White arrows indicate AIS start and end positions. Scale bar: 50 μm. (C) Quantification of AIS length change in response to optogenetic stimulation. (D) Quantification of final AIS length following stimulation pooled from 3 independent experiments: wild type (n = 27), iso-TDP-43^G298S^ (n = 27), TDP-43^G298S^ (n = 27), and C9orf72-3 (n = 21). (E) Whole-cell current clamp recordings pooled from 3 independent experiments showing current and voltage thresholds for 2 week wild-type (n = 21), iso-TDP-43^G298S^ (n = 28), TDP-43^G298S^ (n = 28), and C9orf72-3 (n = 17) MNs and with 3 h 20 Hz 488 nm optogenetic stimulation: wild type (n = 15), iso-TDP-43^G298S^ (n = 20), TDP-43^G298S^ (n = 17), and C9orf72-3 (n = 12). Poststimulation wild-type and C9orf72 recordings pooled from 2 independent experiments. Current threshold two-way ANOVA: effect of genotype ****p ≤ 0.0001, effect of stimulation ***p = 0.0005. Voltage threshold two-way ANOVA: effect of genotype **p = 0.0018, effect of stimulation ns (not significant). (F) Relationship between injected current and firing frequency with and without optogenetic stimulation taken from current clamp recordings; representative AP firing traces at 100 pA current injection. Error bars represent the SEM. p values from one-way ANOVA tests with Tukey’s comparison (D), unpaired t tests (C), or multiple unpaired t tests (F). *p < 0.05, **p < 0.01, ***p < 0.001, and ****p < 0.0001. See also Figure S2 and Table S1.

Activity-dependent shortening of the AIS is a mechanism by which neurons reduce Na+ conductance and intrinsic excitability in response to elevated neuronal activity to maintain network activity within a set range.^22,24^ We wanted to understand whether AIS plasticity in response to elevated neuronal activity could be dysfunctional in the ALS human induced pluripotent stem cell (hiPSC) MNs. We found that AIS length in early wild-type and isogenic control hiPSC MNs was strongly reduced in response to short-term optogenetic stimulation (bursts of 20 Hz stimuli delivered with a 1 s interval for 3 h), whereas AIS length did not significantly change in *TDP-43*^*G298S*^ or *C9orf72-3* MNs following stimulation (Figures 2A–2C). As a result, the difference in AIS length between control and mutant MNs already present in baseline conditions (Figures 1B–1D) was even more pronounced after stimulation (Figure 2D). The proportion of neurons responsive to optogenetic stimulation was comparable across lines, and optogenetic stimulation had no effect on other neuronal parameters including dendrite number and cell size (Figure S2E). Consistent with AIS shortening, we found that the current threshold for AP initiation was significantly increased in both the wild-type and isogenic control MNs following optogenetic stimulation but was not significantly altered in *TDP-43*^*G298S*^ and *C9orf72-3* MNs (Figure 2E). Furthermore, while voltage thresholds under baseline conditions were not significantly different between control and ALS hiPSC MNs, we observed diverging voltage thresholds following optogenetic stimulation, further contributing to abnormal homeostatic regulation of intrinsic excitability (Figure 2E). Finally, we observed a significant decrease in the maximum firing frequency when plotting the relationship between injected current and AP spiking in wild-type and isogenic control MNs but not in *TDP-43*^*G298S*^ and *C9orf72-3* MNs following optogenetic stimulation (Figure 2F). Again, these differences in excitability could not be explained by changes in passive electrical properties (Table S1). Taken together, our findings show that AIS plasticity is impaired in ALS-related MNs, a feature that leads to abnormal homeostatic regulation of intrinsic neuronal excitability.

### Hyperexcitable early ALS patient-derived MNs drive spontaneous myofiber contractions of *in vitro* human motor units

**Figure 3:**
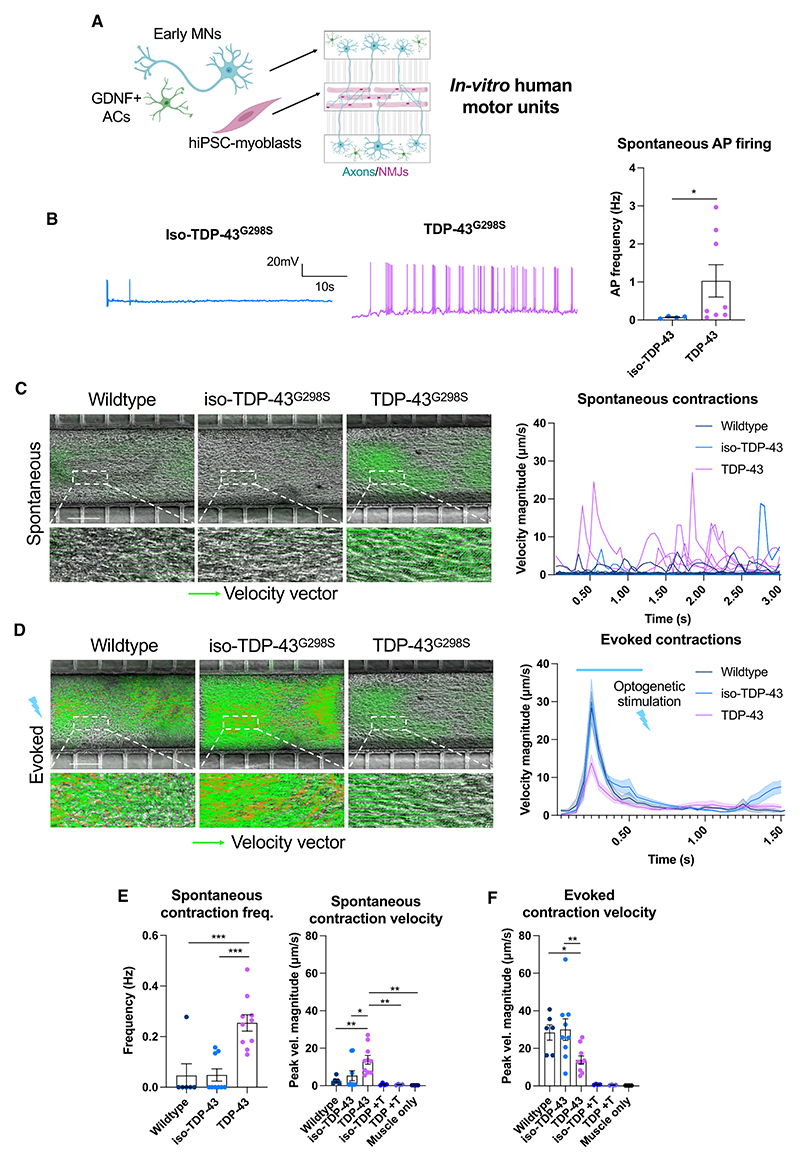
Hyperexcitable early (2 week) ALS motor neurons drive spontaneous myofiber contractions (A) Schematic showing generation of neuromuscular co-cultures in compartmentalized microdevices. (B) Spontaneous AP firing frequency taken from passive membrane recordings of all spontaneously firing neurons. (C) Particle image velocimetry (PIV) analysis of spontaneous myofiber contractions in co-cultures pooled from 3 independent experiments, containing wild-type (n = 6), iso-TDP-43^G298S^ (n = 9), and TDP-43^G298S^ (n = 10) MNs and overlayed spontaneous contraction traces. Velocity vectors shown in green. Scale bar: 200 μm. (D) PIV analysis of optogenetically evoked myofiber contractions and combined traces of optogenetically evoked myofiber contractions. (E) Quantification of spontaneous myofiber contraction frequency and velocity with and without addition of the AChR blocker d-tubocurarine (T) and in muscle-only controls. (F) Quantification of optogenetically evoked myofiber contraction velocity with and without addition of d-tubocurarine and in muscle-only controls. Error bars represent the SEM. p values from one-way ANOVA tests with Tukey’s comparison (E and F). Unpaired t test used for (B). *p < 0.05, **p < 0.01, ***p < 0.001, and ****p < 0.0001. See also Figure S3 and Videos S1 and S2.

We wanted to understand how impaired homeostatic regulation of intrinsic excitability would impact motor unit function. In the early stages of ALS, motor units are hyperexcitable, leading to spontaneous muscle contractions or fasciculations^9^ that have been shown to correlate with increased disease severity and reduced survival.^9,13^ We generated an *in vitro* hiPSC-derived motor unit model using compartmentalized microdevices^28^ (Figure 3A) and focused our analysis on *TDP-43*^*G298S*^ MNs and isogenic controls. In conjunction with an increase in AIS length and neuronal excitability, *TDP-43*^*G298S*^ MNs displayed increased spontaneous AP firing (Figure 3B), in line with previous studies.^22,29^ Consistent with this, motor units comprising TDP-43^G298S^ MNs and wild-type muscle displayed an increase in both the frequency and velocity of spontaneous myofiber contractions (Figures 3C and 3E; Video S1). Myofibers were wild type across all conditions, demonstrating that increased spontaneous neuromuscular activity is specific to the intrinsic properties of the innervating MNs. To confirm this, we blocked neuromuscular transmission using the acetylcholine receptor (AChR) agonist d-tubocurarine (DTC), which abolished spontaneous contractions to levels observed in myofiber-only controls (Figure 3E). Motor unit functionality was confirmed through optogenetic stimulation of MNs, which induced robust myofiber contractions that could be abolished by DTC treatment (Figure S3A). Interestingly, ALS hiPSC motor units displayed a reduction in maximum optogenetically evoked contraction velocity relative to controls (Figures 3D and 3F; Video S2), which was linked to a reduction in axonal outgrowth and number of neuromuscular junctions (NMJs) (Figures S3B and S3C), despite initial axon outgrowth being normal (Figure S3D). Axon coverage was also affected in MN-only cultures, and this difference between control and TDP-43 mutant MNs became more pronounced between weeks 2 and 4 (Figure S3E). Taken together, these data show the simultaneous occurrence of motor unit hyperexcitability and loss of axon outgrowth and functional neuromuscular synapses in an *in vitro* hiPSC model of ALS motor units.

### Late ALS patient-derived MNs progress to a shorter AIS and hypoexcitability

**Figure 4:**
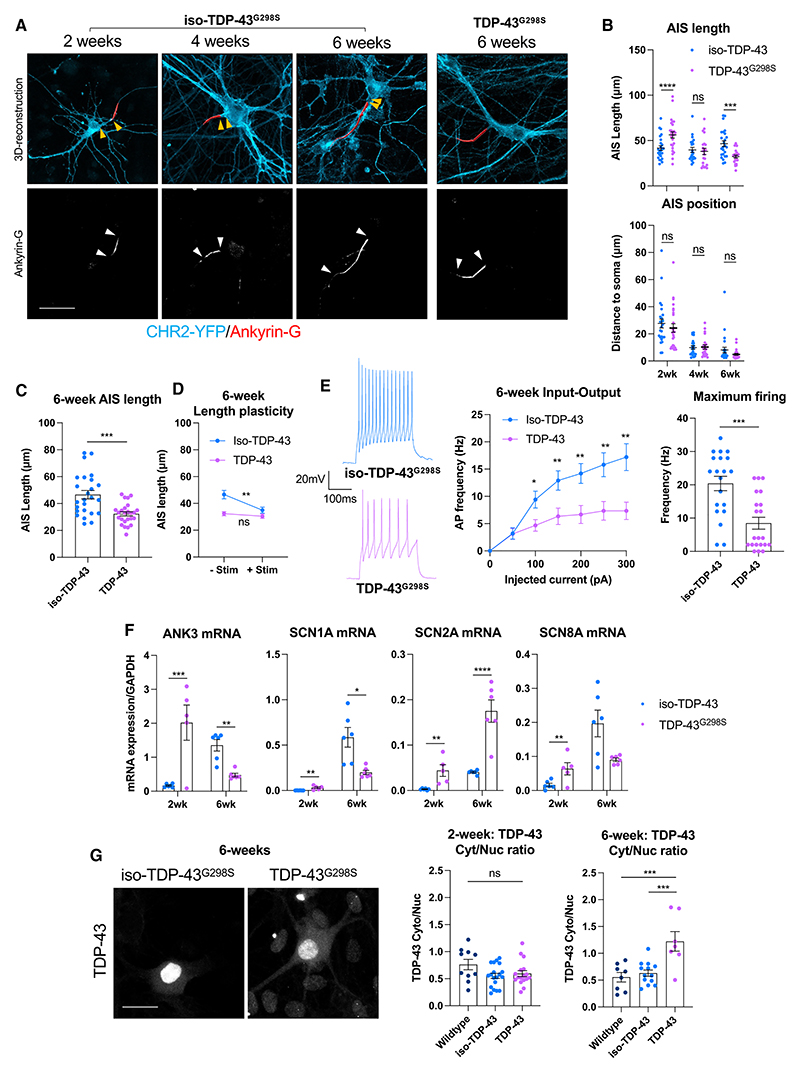
Late human ALS motor neurons progress to a shorter AIS and hypoexcitability (A) Reconstructions of the AIS (red) in 2, 4, and 6 iso-TDP-43^G298S^ and TDP-43^G298S^ MNs with and without 3 h 5 Hz optogenetic stimulation. Based on AnkG immunofluorescence staining, counterstained against CHR2-YFP (cyan). White arrows indicate AIS start and end positions, and orange arrows indicate distance from AIS start to the soma. Scale bar: 50 μm. (B) Quantification of AIS length and position pooled from 3 independent experiments in 2 (n = 25), 4 (n = 21), and 6 week (n = 25) iso-TDP-43^G298S^ and 2 (n = 26), 4 (n = 21), and 6 week (n = 25) TDP-43^G298S^ MNs. (C) Quantification of AIS length based on AnkG staining in baseline unstimulated conditions at 6 weeks. (D) Quantification of AIS length plasticity at 6 weeks. Iso-TDP-43^G298S^ plus stimulation (n = 25), TDP-43^G298S^ plus stimulation (n = 25). (E) Relationship between injected current and firing frequency pooled from 3 independent experiments taken from whole-cell current clamp recordings of 6 week iso-TDP-43^G298S^ (n = 20), and TDP-43^G298S^ (n = 21) MNs, and maximum evoked AP firing frequency (Hz) taken from current clamp recordings. (F) RT-qPCR analysis of *ANK3* (Ankyrin-G), *SCN1A* (Nav1.1), *SCN2A* (NaV1.2), and *SCN8A* (Nav1.6) expression in early (2 week) and late (6 week) MNs. (G) Immunofluorescence of TDP-43 in 6 week MNs. Scale bar: 25 μm. Quantification of the cytoplasmic-to-nuclear ratio of TDP-43 fluorescence intensity. Error bars represent the SEM. p values from one-way ANOVA with Tukey’s multiple comparisons test (G), multiple unpaired t tests (E), and unpaired t tests (B–D and F). *p < 0.05, **p < 0.01, ***p < 0.001, and ****p < 0.0001. See also Figures S1 and S4 and Table S1.

While neuronal hyperexcitability is a feature in the early stages of ALS, progression to hypoexcitability has been reported at later stages.^9^ To this end, we cultured *TDP-43*^*G298S*^ and isogenic control MNs for up to 6 weeks postenrichment and measured AIS and intrinsic excitability properties. We observed a switch from a longer AIS to a shorter AIS in the late ALS hiPSC MNs relative to isogenic controls (Figures 4A–4C). This was accompanied by impaired activity-dependent AIS plasticity in response to optogenetic stimulation (Figure 4D). In conjunction with the observed AIS shortening, *TDP-43*^*G298S*^ MNs progressed to hypoexcitability, characterized by a downward shift in the relationship of injected current to AP firing, as well as a reduction in maximum AP firing relative to isogenic controls (Figure 4E). Dynamic changes to the inward and outward Na+ and K+ currents and to AP properties were also observed between the TDP-43^G298S^ and isogenic control neurons at these different stages (Figures S4A–S4E). Expression of key AIS genes including the AIS master scaffolding protein ankyrin-G (*ANK3*) and voltage-gated sodium channels responsible for AP generation, Nav1.1 (*SCN1A*), Nav1.2 (*SCN2A*), and Nav1.6 (*SCN8A*), mirrored this dynamic switch from a longer to a shorter AIS in early versus late MNs, except for Nav1.2 (*SCN2A*), which always showed a chronic increase in expression in *TDP-43*^*G298S*^ MNs (Figure 4F). Finally, we also found that *TDP-43*^*G298S*^ MNs began to develop TDP-43 pathology at this later stage, characterized by reduced nuclear TDP-43 and increased cytoplasmic TDP-43, although we did not observe pathological aggregates (Figures 4G and S4F). To further validate these findings, we analyzed a recently published ALS postmortem spinal cord RNA sequencing (RNA-seq) dataset and found reduced expression of *ANK3*, as well as mis-regulation of other AIS genes, such as *KCNQ2* and *SCN1A* (Figure S1C).^30^ In addition, we optimized ankyrin-G staining of ventral horn spinal MNs in postmortem tissue (Figure S1D) and found that AIS length was reduced in sporadic ALS and TDP-43-associated ALS cases compared to control cases (Figures S1E–S1G), supporting our *in vitro* data showing a switch from a longer to a shorter AIS in late ALS hiPSC MNs.

## Discussion

Our study shows that increased AIS length and impaired AIS plasticity is an important mechanism driving abnormal regulation of neuronal activity and intrinsic hyperexcitability in early ALS hiPSC MNs from diverse TDP-43 and C9orf72 backgrounds. In turn, these hyperactive MNs triggered an increase in spontaneous myofiber contractions of *in vitro* hiPSC ALS motor units, mirroring muscle fasciculations in patients with ALS. Conversely late hiPSC MNs and postmortem ALS spinal MNs displayed a switch to AIS shortening, and hiPSC MNs progressed to neuronal hypoexcitability. At all stages, expression of the AIS master scaffolding protein *ANK3* and key AIS-specific voltage-gated sodium channels involved in AP generation mirrored these dynamic changes in AIS function. Mis-regulation of AIS gene expression was also found in postmortem ALS spinal MNs. Taken together, our findings point toward an important and dynamic role of the AIS as a site of dysfunction in ALS MNs.

AIS length is a crucial determinant of Na+ conductance during AP initiation and an integral mechanism by which neurons set their intrinsic excitability and AP threshold.^20–22,24^ We found that ALS hiPSCs MNs displayed an increase in the length of the AIS and a corresponding increase in neuronal excitability characterized by a reduction in current threshold for AP generation, a shift in the input-output relationship, and an increase in the maximum firing rate of MNs (Figure 1). Structural plasticity of the AIS in response to elevated neuronal activity was also substantially impaired in these ALS hiPSC MNs (Figures 2 and 4). Previous studies have shown a remarkable capacity for the AIS to change its length in response to altered neuronal activity—a homeostatic mechanism that is thought to either elevate or dampen intrinsic excitability to prevent abnormal levels of network activity.^22,24,25,31^ Mathematical models exploring the functional outcome of changes in AIS geometry have notably shown that length is an important factor in determining neuronal excitability and spontaneous firing.^20,21,29^ In ALS hiPSC MNs, AIS plasticity in response to optogenetically induced activity was substantially impaired, leading to an even greater disparity in AIS length following stimulation, beyond that observed in baseline conditions. This led to an impaired ability of MNs to homeostatically regulate their intrinsic excitability. Indeed, this impaired plasticity may contribute to the increase in AIS length in baseline conditions since neurons would not be able to adapt to ongoing fluctuations in network activity, leading to a chronic extension of the AIS.

Such changes to AIS function may underpin the changes in neuronal excitability that have previously been reported in patients, as well as in mouse and hiPSC models of the disease,^10,12,32–37^ and may also explain why structural changes to the AIS are seen in postmortem patient spinal cord (Figures S1E–S1G).^38^ In the mutant SOD1 mouse model, symptomatic mice also display increased AIS length and an increased sodium current in MNs,^39^ while a previous study by the same group showed that presymptomatic mice display a shortening and widening of the AIS.^40^ Our data would also suggest stage-specific changes to AIS function and neuronal activity (Figure 4).

Indeed, we observe that substantial changes to AIS function and excitability occur prior to the onset of overt TDP-43 pathology and could represent early changes to neuronal function that de-stabilize and increase the vulnerability of MNs to further pathological processes. Elevated neuronal activity caused by altered AIS structure and impaired plasticity could contribute to TDP-43- and C9orf72-related pathological processes. Recent work has shown that elevated neuronal activity up-regulates shortened TDP-43 isoforms that accumulate in the cytoplasm and sequester full-length TDP-43 to form insoluble inclusions.^41^ Furthermore, neuronal hyperactivity has been shown to increase repeat-associated non-AUG (RAN) translation of C9orf72 dipeptide repeats (DPRs).^42^ As such, a cycle could develop whereby hyperexcitability contributes to TDP-43- and C9orf72-related pathological processes, which in turn further dysregulates neuronal excitability. It is possible that this early hyperexcitability may partly contribute to the TDP-43 mis-localization observed in the later MNs (Figure 4).

Furthermore, impaired capacity to adapt and homeostatically regulate AIS length and intrinsic excitability in lower MNs would lead to elevated activity and increased spontaneous AP firing,^22,29^ possibly driving spontaneous contractions of innervated muscle seen in patients.^9^ In line with this, we found that ALS MNs fired more spontaneous APs, triggering an increase in the frequency and velocity of spontaneous myofiber contractions in co-cultures of ALS MNs and hiPSC-derived myoblasts. Increased muscle fas-ciculations in patients with ALS have been shown to correlate with increased disease severity and reduced survival.^13^ Furthermore, riluzole, one of the only approved drugs to treat ALS, acts to dampen neuronal excitability mainly by inhibiting TTX-sensitive sodium channels—suggesting that modulation of neuronal excitability via Na+ conductance can affect disease outcomes in patients.^14–16^ Other than potential effects on TDP-43- and C9orf72-related pathology, elevated neuronal activity may also drive excitotoxicity, characterized by impaired calcium buffering, generation of reactive oxygen species, and formation of the mitochondrial permeability transition pore that further dysregulates Ca2+ buffering. In turn, this is thought to activate calpain, cleaving the apoptosis-inducing factor (AIF) and ultimately inducing neuronal apoptosis and death.^43^ This hyperexcitability-driven excitotoxicity may affect motor unit vulnerability. Indeed, we observed a reduction in axonal outgrowth and NMJ number in the hyperactive ALS hiPSC motor units, which led to a reduction in optogenetically evoked myofiber contraction velocity. This resembles the simultaneous occurrence of muscle fasciculations and muscle weakness observed in the early stages of ALS.^9^ It is possible that motor unit dysfunction may further destabilize the AIS and neuronal excitability since it has been shown that blockage of the NMJ either pharmacologically or through axotomy leads to elongation of the AIS and increased excitability of MNs.^44,45^ As such, a delicate interplay between the AIS, the NMJ, and neuronal excitability may be an important focus for axonal dysfunction in ALS MNs.

Generally, at a cellular and circuit level, it will be important to understand the relationship between alterations in synaptic function and intrinsic excitability, in the context of both the fasciculations observed in lower MNs as well in the cortical hyper-excitability seen in ALS-frontotemporal dementia (FTD).^46^ Numerous studies have reported loss of synaptic transmission in ALS models, and recent work has found aberrant splicing and loss of the crucial synaptic protein UNC13A upon TDP-43 depletion, likely driving substantial impairments in synaptic transmission and plasticity.^32,47,48^ Our work has found that both synaptic and intrinsic excitability phenotypes do indeed co-exist (Figures 1 and 3). Future work should aim to understand how synaptic changes, combined with altered AIS properties and intrinsic hyperexcitability, affects network-level function of both lower MNs and cortical networks, as well as the temporal order in which these events take place.

Beyond its impact on neuronal excitability, abnormal AIS function could also contribute to other aspects of cellular pathology in ALS, most notably in axonal transport.^49^ The AIS is known to act as an important cargo transport filter between somatic and axonal compartments.^50^ Recent work has shown that the AIS in *SOD1*^*G93A*^ mice fails to disassemble in response to axonal damage, which blocks the anterograde influx of mitochondria required for nerve regeneration.^51^ Abnormal AIS function may, therefore, not only play an important role in destabilization of neuronal excitability but also in dysregulation of axonal transport and cargo trafficking.

We also observed a switch to AIS shortening and hypoexcitability in the later hiPSC MNs and in postmortem ALS spinal cord MNs (Figures 4 and S1E–S1G). Such a biphasic change in neuronal activity has been observed in patients with ALS as well as various mouse and hiPSC model systems.^9,35,36,52^ Interestingly, we found that expression of the AIS master scaffolding protein ankyrin-G (*ANK3*) and voltage-gated sodium channels Nav1.1 (*SCN1A*) and Nav1.6 (*SCN8A*) mirrored this dynamic switch in AIS length and excitability in ALS MNs. *ANK3* expression was also downregulated in postmortem ALS spinal cords (Figure S1C) and was associated with a reduction in AIS length measured by ankyrin-G staining (Figures S1E–S1G), in line with previously published data.^38^ It is likely that onset of TDP-43 mis-localization in the later neurons partly contributes to this switch in AIS length through changes in gene expression. Indeed, TDP-43 has been found to bind and modulate splicing and expression of various AIS genes,^53^ and abnormal expression patterns can be seen in numerous RNA-seq datasets.^6,30,47,48^ These include genes integral to AIS cytoskeletal organization such as *ANK3* and *ANK2*, those involved in AP initiation and modulation such as *SCN1A, SCN2A*, and *KCNQ2*, and those involved in AIS plasticity such as *CACNA1E* and *CAMK2B*. Future work should seek to further understand how progression of TDP-43 pathology impacts splicing, stability, transport, translation, and localization of AIS genes controlling neuronal excitability.

In summary, we show that the AIS is an important site of dysfunction in ALS hiPSC MNs. We found that structural, functional, and molecular changes to the AIS—the site of AP initiation—led to dynamic dysfunction of neuronal excitability, providing mechanistic insights into the altered activity of MNs and motor units in ALS.

### Limitations of the study

In the present study, only hiPSC MNs carrying mutations in the genes *TDP-43* and *C9orf72* were investigated. Therefore, our conclusions regarding abnormal AIS length and homeostatic plasticity are limited to these genotypes. More specifically, some forms of familial ALS, such as the ones caused by mutations in the genes *FUS* and *SOD1*, do not show TDP-43 pathology, and in these cases, abnormal MN excitability (and other phenotypes) is likely to be caused by different molecular and cellular pathways. Furthermore, in our current models, only the MNs carry ALS-linked mutations, whereas astrocytes and myofibers have wild-type genotypes. The impact of non-autonomous effects on the AIS, caused by interactions of mutant glia or myofibers with MNs, will have to be determined in future studies.

The postmortem data we present are from a small-scale analysis owing to a lack of tissue availability and poor quality of the late-stage ALS spinal cord tissue obtained. Having optimized AIS staining in human spinal cord tissue, we hope that this published approach will enable future studies to expand on this work by analyzing the AIS across more postmortem cases, different spinal cord and brain regions, and in neurons with and without TDP-43 pathology.

## Star★Methods

Detailed methods are provided in the online version of this paper and include the following:

- KEY RESOURCES TABLE
- RESOURCE AVAILABILITY
  ○ Lead contact
  ○ Materials availability
  ○ Data and code availability
- METHOD DETAILS
  ○ Engineering hiPSC-lines
  ○ Cell culture and differentiation
  ○ Magnetic activated cell sorting (MACS)
  ○ Targeted gene editing
  ○ Immunocytochemistry
  ○ RT-qPCR
  ○ Western blot
  ○ Electrophysiology
  ○ Microdevice fabrication and neuromuscular co-cultures
  ○ Optogenetic stimulation
  ○ Postmortem AIS staining
  ○ Analysis of AIS morphology
  ○ Analysis of axon outgrowth and detection of NMJs
  ○ Particle image velocimetry (PIV) analysis of myofiber contractions
  ○ Analysis of electrophysiological measurements
- QUANTIFICATION AND STATISTICAL ANALYSIS

## Star★Methods

### Key Resources Table

**Table:** 

REAGENT or RESOURCE	SOURCE	IDENTIFIER
Antibodies		
Mouse anti-Ankyrin-G	NeuroMab	Cat# N106/36; RRID:AB_10673030
Rabbit anti-GFP	Thermo Fisher Scientific	Cat# A11122; RRID:AB_221569
Rabbit anti-ISL1	Abcam	Cat# ab109517; RRID:AB_10866454
Mouse anti-TUBB3	R&D Systems	Cat# MAB1995; RRID:AB_357520
Mouse anti-Synaptic vesicle glycoprotein 2A (SV2A)	DSHB	Cat# SV2; RRID:AB_2315387
Rat anti-Acetylcholine receptor nicotinic alpha 1 subunit (CHRNA1)	DSHB	Cat# mAb35; RRID:AB_528405
Mouse anti-Titin	DSHB	Cat# 9D10; RRID:AB_528491
Mouse anti-CD14	ATCC	Cat# HB-246; RRID:AB_2782995
Mouse anti-Choline acetyltransferase (CL3173)	Novus Biologicals	Cat# NBP2-46620 RRID: AB_2922998
Goat anti-Mouse IgG2a, Alexa Fluor 647	Thermo Fisher Scientific	Cat# A-21241; RRID:AB_141698
Goat anti-Mouse IgG2a, Alexa Fluor 488	Thermo Fisher Scientific	Cat# A-21131; RRID:AB_2535771
Goat Anti-Mouse IgG, Alexa Fluor 488	Thermo Fisher Scientific	Cat# A-32723; RRID:AB_2633275
Donkey anti-Rabbit IgG, Alexa Fluor 488	Thermo Fisher Scientific	Cat# A-21206; RRID:AB_2535792
Donkey anti-Rabbit IgG, Alexa Fluor 647	Thermo Fisher Scientific	Cat# A-31573; RRID:AB_2536183
Goat anti-Mouse IgM, Alexa Fluor 405	Abcam	Cat# ab175662
Goat anti-Mouse IgG1, Alexa Fluor 647	Thermo Fisher Scientific	Cat# A-21240; RRID:AB_2535809
Goat anti-Rat IgG, Alexa Fluor 555	Thermo Fisher Scientific	Cat# A-21434; RRID:AB_141733
Goat anti-mouse IgG, biotinylated	Vector Laboratories	Cat# BA-9200 RRID:AB_2336171
Mouse anti-Human TDP-43	R&D Systems	Cat# MAB7778
Rabbit anti-Human TDP-43 (Western Blot)	Proteintech	Cat# 10782-2-2AP RRID:AB_615042
Rabbit anti-Human Vinculin (Western Blot)	Proteintech	Cat# 26520-1-AP RRID:AB_2868558
Goat anti-Mouse IgG MicroBeads	Miltenyi Biotech	Cat# 130-048-402 RRID:AB_244361
Chemicals, peptides, and recombinant proteins		
DAPI	Sigma-Aldrich	Cat# D9542
Human Laminin 521, recombinant	Biolamina	Cat# LN521-05
StemMACS™ iPS-Brew XF medium	Miltenyi Biotech	Cat# 130-104-368
TrueCut CAS9 protein v2	Thermo Fisher Scientific	Cat# A36498
Penicillin-Streptomycin 100x	Thermo Fisher Scientific	Cat# 15140122
TrypLE™ Express Enzyme	Thermo Fisher Scientific	Cat# 12605010
Y-27632 (ROCK inhibitor)	Tocris	Cat# 1254
DMEM/F-12 medium	Thermo Fisher Scientific	Cat# 11554546
Neurobasal medium	Thermo Fisher Scientific	Cat# 21103049
MegaCell DMEM	Sigma-Aldrich	Cat# M3942
Horse Serum	Thermo Fisher Scientific	Cat# 16050122
Fetal Bovine Serum	Thermo Fisher Scientific	Cat# 26140079
N-2 supplement	Thermo Fisher Scientific	Cat# 17502001
NeuroBrew-21 supplement	Miltenyi Biotech	Cat# 130-097-263
Collagenase, type IV	Thermo Fisher Scientific	Cat# 17104019
Growth factor reduced-Matrigel	Corning	Cat# 354230
L-Glutamine (200 mM)	Thermo Fisher Scientific	Cat# 25030081
CHIR 99021 (GSK3 inhibitor)	Tocris	Cat# 4423
SB 431542 (TGFb type 1-receptor inhibitor)	Tocris	Cat# 1614
LDN 193189 (SMAD inhibitor)	Stemgent	Cat# 04-0074-base
Retinoic acid	Sigma-Aldrich	Cat# R2625
Purmorphamine (SHH agonist)	Tocris	Cat# 4551
Valproic acid	Stemgent	Cat# 04-0007
Accutase	Stem Cell Technologies	Cat# 07920
Advanced DMEM/F-12 medium	Thermo Fisher Scientific	Cat# 12634028
β-mercaptoethanol 1000x	Thermo Fisher Scientific	Cat# 21985023
B-27 supplement	Thermo Fisher Scientific	Cat# 17504044
Smoothened agonist, SAG	Sigma-Aldrich	Cat# 566660
Trypsin/EDTA solution	Thermo Fisher Scientific	Cat# 25200056
DNase I	Roche	Cat# 10104159001
Poly-L-Ornithine solution 0.01%	Sigma-Aldrich	Cat# A004M
Mouse Laminin	Thermo Fisher Scientific	Cat# 23017015
MEM Non-essential amino acid solution 100x	Thermo Fisher Scientific	Cat# 11140050
DAPT (Notch inhibitor)	Stem Cell Technologies	Cat# 72082
Human BDNF, recombinant	Peprotech	Cat# 450-02
Human GDNF, recombinant	Peprotech	Cat# 450-10
Human FGF2, recombinant	R&D Systems	Cat# 233-FB
Doxycycline hyclate	Sigma-Aldrich	Cat# D5207
Low-glucose DMEM medium	Thermo Fisher Scientific	Cat# 11885084
Bovine serum albumin fraction V solution 7.5%	Thermo Fisher Scientific	Cat# 11500496
Norland Optical Adhesive 73	Norland Products	Cat# NOA73
Lipidure powder	Amsbio	Cat# AMS.52000034GB1G
d-Tubocurarine	Sigma-Aldrich	Cat# T2379
Antioxidant supplement 1000x	Sigma-Aldrich	Cat# A1345
Fibrinogen (from bovine plasma)	Sigma-Aldrich	Cat# F8630
Thrombin (from bovine plasma)	Sigma-Aldrich	Cat# 112374
Dimethyl sulfoxide, DMSO	Sigma-Aldrich	Cat# D2438
Paraformaldehyde solution 16%	Thermo Fisher Scientific	Cat# 043368.9M
Protein Assay Dye Reagent Concentrate	BioRad	Cat# 5000006
Protease inhibitor cocktail	Sigma-Aldrich	Cat# P2714
VectaShield Antifade Mounting Medium	Vector Laboratories	Cat# H-1000
3,3’-Diaminobenzidine (DAB)	DAKO	Cat# K3465
Critical commercial assays		
PureLink RNA Mini Kit	Thermo Fisher Scientific	Cat# 12183018A
GoScript™ Reverse Transcriptase kit	Promega	Cat# A5001
SYBR Green PCR Master Mix	Thermo Fisher Scientific	Cat# 4309155
Clarity Western ECL Substrate	BioRad	Cat# 170506
Experimental models: Cell lines		
PAMV1 human iPSC line	HIPSCI	https://www.hipsci.org/lines/#/lines/HPSI1013i-pamv_1
H9 human ESC line	WiCell	https://www.wicell.org/home/stem-cells/catalog-of-stem-cell-lines/wa09.cmsx
Becker S6 C9orf72-mutant human iPSC line (C9-1)	Mehta et al.^54^ Prof S. Chandran, University of Edinburgh	Available from University of Edinburgh
Becker S6 isogenic control human iPSC line (C9-1Δ)	Mehta et al.^54^ Prof S. Chandran, University of Edinburgh	Available from University of Edinburgh
M211R2 C9orf72-mutant human iPSC line (C9-3)	Mehta et al.^54^ Prof S. Chandran, University of Edinburgh	Available from University of Edinburgh
DN1qV4 C9orf72-mutant human iPSC line (C9-2)	Mehta et al.^54^ Prof S. Chandran, University of Edinburgh	Available from University of Edinburgh
DN1qV4 isogenic control human iPSC line (C9-2Δ)	Mehta et al.^54^ Prof S. Chandran, University of Edinburgh	Available from University of Edinburgh
TDP-43(G298S)-mutant human iPSC line	Barton et al.^55^ Prof S. Chandran, University of Edinburgh	Available from University of Edinburgh
TDP-43(G298S) isogenic control human iPSC line	This paper	Available from KCL. Additional MTA with University of Edinburgh is required.
PAMV1 human iPSC line, Tet-ON:: PAX7 (iPAX7) transgenic	Cheesbrough et al.^56^	Available from KCL. Additional MTA with the Wellcome Sanger Institute is required.
H9 human ESC line, Hb9:CD14/CAG:: ChR2-YFP transgenic	This paper	Available from KCL. Additional MTA with WiCell is required.
TDP-43(G298S)-mutant human iPSC line, Hb9:CD14/CAG::ChR2-YFP transgenic	This paper	Available from KCL. Additional MTA with University of Edinburgh is required.
TDP-43(G298S) isogenic control human iPSC line, Hb9:CD14/CAG::ChR2-YFP transgenic	This paper	Available from KCL. Additional MTA with University of Edinburgh is required.
M211R2 C9orf72-mutant human iPSC line, Hb9:CD14/CAG::ChR2-YFP transgenic	This paper	Available from KCL. Additional MTA with University of Edinburgh is required.
IB10 mouse ESC line, GFAP::CD14/CAG:: GDNF transgenic	Machado et al.^57^	Available from KCL.
Oligonucleotides		
sygRNA	Sigma-Aldrich	Cat# TRACRRNAMOD-5NMOL
sgRNA	Sigma-Aldrich	GGATTTGGTAATAGCAGAGGGGG
ANK3 forward primer	Sigma-Aldrich	AAAGGACTGCCTCAAACAGCGG
ANK3 reverse primer	Sigma-Aldrich	CTAAGGATGCGAAGCTCTGTCG
SCN1A forward primer	Sigma-Aldrich	GGACTGTATGGAGGTTGCTGGT
SCN1A reverse primer	Sigma-Aldrich	GCAAGGTTGTCTGCACTAAATGAG
SCN2A forward primer	Sigma-Aldrich	CTAGCCTCACTGTGACAGTACC
SCN2A reverse primer	Sigma-Aldrich	TCAACCGTGCTGCCTTCAGATG
SCN8A forward primer	Sigma-Aldrich	GGATTGAGACCATGTGGGACTG
SCN8A reverse primer	Sigma-Aldrich	ATCTGTGGCAGCCAGGTTGTCT
GAPDH forward primer	Sigma-Aldrich	GTCTCCTCTGACTTCAACAGCG
GAPDH reverse primer	Sigma-Aldrich	ACCACCCTGTTGCTGTAGCCAA
Recombinant DNA		
hAAVS1 TALEN Right	Addgene	RRID:Addgene_52342
hAAVS1 TALEN Left	Addgene	RRID:Addgene_52341
AAVS1-Hb9:CD14	This paper	RRID:Addgene_ 204344
PB-CAG::ChR2-YFP	This paper	RRID:Addgene_ 204345
CLYBL-TO-PAX7	Cheesbrough et al.^56^	RRID:Addgene_ 204346
pCAGG-PBase	Wang et al.^58^ Dr P. Liu, Wellcome Sanger Institute	Available from the Wellcome Sanger Institute, Cambridge, UK.
Software and algorithms		
MATLAB R2021A	Mathworks	https://www.mathworks.com/products/matlab.htmlRRID:SCR_001622
PIVlab 2.53 (MATLAB plug-in)	Thielicke & Stamhuis^59^	https://pivlab.blogspot.com/
FIJI (ImageJ)	NIH	https://imagej.net/software/fiji/ RRID:SCR_002285
Imaris 9.2.1	Oxford Instruments	https://imaris.oxinst.com/products/imaris-for-cell-biologists RRID:SCR_007370
Prism 9	GraphPad	https://www.graphpad.com/features RRID:SCR_002798
FlowJo	BD Bioscience	https://www.fiowjo.com/solutions/fiowjo RRID:SCR_008520

## Resource Availability

### Lead contact

Further information and requests for resources and reagents should be directed to and will be fulfilled by the lead contact, Ivo Lieberam (ivo.lieberam@kcl.ac.uk).

### Materials availability

- Plasmids generated in this study have been deposited to Addgene (204344, 204345, 204346).
- Mouse and human stem cell lines generated in this study are available from the lead contact with a completed materials transfer agreement. In some cases, an additional materials transfer agreement with the institution that generated the parental stem cell line will be required (see key resources table).

## Method Details

### Engineering hiPSC-lines

Patient-derived hiPSC lines harboring the pathogenic *TDP-43*^*G298S*^ and *C9orf72-3 (M221R2/C9-3)* mutations were provided by Agnes Nishimura (King’s College London) and Christopher Shaw (King’s College London), and originated from the group of Siddarthan Chandran (The University of Edinburgh). In addition, *C9orf72*-mutant hiPSC lines ‘Becker S6’ (C9-1) and ‘DN1qV4’ (C9-2), as well as the corresponding isogenic control hiPSC lines, were directly obtained from the Chandran group. The *TDP-43*^*G298S*^ line was originally published in.^55^ All *C9orf72*-mutant and control hiPSC lines used in this study have been published in.^54,60^ The wildtype hESC H9 line was acquired from WiCell (Madison. Wisconsin, USA) under a license from the steering committee for the UK Stem Cell Bank (No. SCS11-06).

hiPSC and hESC clones intended for MN differentiation and enrichment were engineered to express the *HB9::hCD14* MACS sortable construct using TALENS based insertion into the AAVS1 safe-harbour locus (Figure S2D)^61^ using a NEPA-21 electroporator (Sonidel). Cell lines were also engineered to express the optogenetic actuator transgene *CAG::CHR2-YFP* using a PiggyBAC-mediated integration system via electroporation. Fluorescence-activated cell sorting (FACS) was carried out using a BD FACSAria 3 (BD Biosciences) to select CHR2-YFP positive cells to generate polyclonal cell lines with comparable YFP expression (Figure S2E). Inducible iPAX7 hiPSC-lines to forward program hiPSCs into myoblast progenitors were generated by TALENS based integration the Doxycycline-inducible PAX7 construct^56^ into the CLYBL safe-harbour locus^62^ of the publicly available HIPSCI line PAMV1, via electroporation.

### Cell culture and differentiation

hiPSCs/hESCs were maintained on 0.4 μg/cm^2^ LN521 basement matrix (BioLamina) in StemMACS iPS-Brew XF with 2% (v/v) iPS-Brew supplement (Miltenyi Biotec) plus 1% (v/v) Penicillin/streptomycin (Gibco). Cells were passaged at 70% confluency as single cells by incubating cells with TrypLE express (Invitrogen) for 5 min at 37°C and replating in 10 μM ROCK inhibitor Y-27632 (Tocris) for 24h.

MN differentiation was based on^63^ with minor modifications. hiPSCs were grown until 100% confluent on 0.4 μg/cm^2^ LN521 basement matrix (BioLamina) in StemMACS iPS-Brew XF medium with 2% (v/v) iPS-Brew supplement (Miltenyi Biotec). Cells were then passaged 1:3 as clumps using 15 min 37°C incubation with 1 mg/mL collagenase IV (Invitrogen) onto plates coated with 1:50 GFR-Matrigel (Corning) in iPS-Brew XF containing 10 μM ROCK inhibitor. After 2–3 days once colonies started to merge media was switched to NEP media (d0), comprising basal media: DMEM/F-12 (Gibco) and Neurobasal (Gibco) in 1:1 mix, with 0.5% (v/v) N-2 supplement (Gibco), 1% (v/v) Neurobrew-21 (Miltenyi Biotec), 2 mM L-Glutamine (Gibco), 1% (v/v) penicillin/streptomycin solution (Gibco), plus 3 μM CHIR99021 (Tocris), 2 μM SB431542 (Tocris) and 0.2 μM LDN193189 (Stemgent) for 6 days. After 6 days cells were again split 1:3 with collagenase IV onto 1:50 Matrigel (BD Biosciences) coated plates and media changed to MNP media, comprising basal media plus 0.1 μM retinoic acid (Sigma-Aldrich), 0.5 μM purmorphamine (Tocris), 1 μM CHIR99021, 2 μM SB431542, 0.2 μM LDN193189. After a further 6 days cells were either frozen or split 1:3 using collagenase IV and switched to MNP expansion media, comprising basal media plus 0.1 μM retinoic acid, 0.5 μM purmorphamine, 3 μM CHIR99021, 2 μM SB431542, 0.2 μM LDN193189, 0.5 μM valproic acid (Stemgent). Cells were passaged up to 2 times in expansion conditions before freezing.

In all experiments with the *C9orf72* lines, in experiments involving electrophysiology recordings following optogenetic stimulation of wildtype MNs, and in about one-third of the AIS length measurements of wildtype, iso-*TDP-43*^*G298S*^ and *TDP-43*^*G298S*^ mutant MNs, an alternative hiPSC-MN differentiation protocol based on embryoid body (EBs) cultures was used.^64^ We confirmed that the AIS and excitability phenotypes we observed were the same with both protocols. Briefly, hiPSC cultures were partially dissociated on day-0 with cold Accutase (Stem Cell Technologies), and detached colonies were resuspended in N2B27 medium (ADMEM/F12 (Gibco) and Neurobasal (Gibco) medium 1:1 mix; 1% (v/v) penicillin/streptomycin; 2 mM L-glutamine; 55 μM β-mercaptoethanol (Gibco); 2% (v/v) B-27 supplement (1x); 1% (v/v) N-2 supplement) and plated in N2B27 medium with 10 μM Y-27632, 20 μM SB431542), 0.1 μM LDN193189 and 3 μM CHIR99021. On day-2, the EBs were passaged and re-plated in the same N2B27 medium with small molecules, except that 0.1 μM retinoic acid and 0.5 μM Smoothened agonist (Merck) was added. The EBs were passaged every 2–3 days until day-9, when they were dissociated with Trysin/EDTA (Gibco) and 25 μg/mL Dnase-I (Roche), and plated at a density of 5×10^4^ on plates pre-coated with poly-L-Ornithine (Sigma) and mouse Laminin (Invitrogen) in N2B27 medium with 1% (v/v) non-essential amino acids (Gibco), 0.1 μM retinoic acid, 0.5 μM Smoothened agonist, 10 μM Y-27632 and 10 μM DAPT. On day-11, the medium was changed and further supplemented with 10 ng/mL BDNF (Peprotech) and 10 ng/mL GDNF (Peprotech). On day-14, the medium was replaced by N2B27 medium with 1% (v/v) non-essential amino acids, 10 ng/mL BDNF and 10 ng/mL GDNF.

Myoblast differentiation was based on^65^ with minor modifications, using the custom built iPAX7 knock-in hiPSC line rather than lentiviral transduction. At 70% confluency hiPSCs were split onto 1:50 Matrigel coated plates at a density of 33k/cm^2^ in iPS-brew XF plus 10 μM ROCK inhibitor Y-27632. The following day media was replaced with E6 medium (Gibco) plus L-2mM glutamine and 1% (v/v) Penicillin/streptomycin and 10 μM CHIR99021 for 2 days, after which CHIR99021 was removed and replaced with 2 μg/mL doxycycline (Sigma) for 18 days with 10 ng/mL FGF2 (R&D Systems) added from day 5. Myoblast progenitors were cultured on 1:50 Matrigel-coated flasks in expansion media, comprising MegaCell DMEM (Sigma-Aldrich), with 5% fetal bovine serum (Gibco), 1% (v/v) non-essential amino acids (Gibco), 2 μM L-glutamine, 1% (v/v) penicillin/streptomycin, 55 μM β-Mercaptoethanol (Gibco) with 2 μg/mL doxycycline and expanded up to 4 passages. To differentiate the myoblasts into myotubes cells were seeded on 1:50 Matrigel-coated plates at a density of 100k/cm^2^ into myogenic differentiation media, comprising low glucose DMEM, 0.5% (v/v) N2 supplement, 2% horse serum (Gibco), 2mM L-glutamine, 1% (v/v) Penicillin/streptomycin. GDNF-expressing mouse ESC astrocyte differentiation was performed as described in.^57^

### Magnetic activated cell sorting (MACS)

Motor neuron progenitors (MNPs) were thawed in MNP differentiation medium, comprising basal medium plus 0.5 μM retinoic acid, 0.1 μM purmorphamine and 10 μM Y-27632 for 24 h at density of 600k/cm^2^ onto Matrigel coated plates. Cells were grown in these conditions for 1 week prior to MACS sorting. Cells were then dissociated in TrypLE with 10 U/ml DNase-I (Roche) for 5–7 min at 37°C into a single cell solution then washed three times in DMEM with 10 U/ml DNase-I. Cells were then filtered using a 40 μm nylon strainer (BD Falcon) and re-suspended in MACS buffer comprising PBS (Gibco), 0.5% (w/v) BSA (ThermoFisher), with 10 U/ml DNase-I and 3 μg/mL anti-CD14 antibody (ATCC), and transferred to a MACSmix tube rotator (Miltenyi Biotec) at 4°C for 15 min. Cells were then washed and resuspended in MACS buffer plus 1:5 anti-mouse IgG microbeads (Miltenyi Biotec) and rotated at 4°C for a further 15 min. Cells were then resuspended in 1 mL MACS buffer and applied to an MS magnetic column mounted to an OctoMACS magnet (Miltenyi Biotec). Cells were washed three times with 500 μL MACS buffer then the positive fraction eluted in 1 mL MACS buffer.

### Targeted gene editing

Targeted gene editing in hiPSCs was achieved using CRISPR-Cas9 mediated homology directed repair to correct the endogenous TDP-43^G298S^ mutation. 3 μg TrueCut CAS9 v2 (ThermoFisher) was combined with 90 μM sygRNA (5′-GGATTTGGTAATAGCA GAGGGGG-3′) (Merck) at RT for 15 min to form an RNP complex. This complex was then electroporated using a NEPA-21 electro-porator (Nepagene) into 1×10^6^ hiPSCs along with 50 μM ddOligo template DNA (ThermoFisher). The donor template was engineered with the GGT codon to correct the AGT codon responsible for the *TDP-43*^*G298S*^ mutation and with a silent Xho1 restriction site in the seed region of the sgRNA. Clones were then screened for the presence of the Xho1 restriction site and positive clones sequenced using Sanger sequencing (SourceBioscience). Subsequently we sequenced ~1000bp upstream and downstream of exon 6 of *TARDBP* and the top 5 predicted off-target sgRNA binding sites to confirm no off-target genome editing. We then carried out G-banding to confirm a normal karyotype (CellGuidanceSystems) (Figure S2B), and Western blot analysis of TDP-43 to confirm normal protein expression (Figure S2C).

### Immunocytochemistry

For imaging, 5000 MACS-sorted MNs were seeded onto 20.000 MACS-sorted GDNF^+^ mouse ESC-derived astrocytes in 96-well high content imaging plates (Greiner 655090) and grown in MN maturation medium, comprising basal medium plus 0.1 μM RA, 0.1 μM purmorphamine and 10 μM Y-27632. Cells were then fixed in 4% (w/v) PFA for 15 min at RT and washed three times in PBS. Subsequently cells were blocked in 3% (w/v) BSA and 0.1% (v/v) Triton X-100 in PBS for 1 h at RT. Neuromuscular co-cultures were additionally permeabilised using 10% (v/v) DMSO. Cells were incubated overnight with primary antibodies in blocking buffer at 4°C. The following primary antibodies and dilutions were used in this study: 1/200 Mouse IgG2a anti-Ankyrin-G (NeuroMab), 1/500 Rabbit anti-GFP (Invitrogen), 1/1000 Rabbit anti-ISL1 (Abcam), 1/1000 Mouse IgG2a anti-TUBB3 (R&D Systems), 1/200 Mouse IgG1 anti-SV2 (DSHB), 1/200 Rat anti-Mab35 (DSHB), 1/20 Mouse IgM anti-Titin (DSHB), and 1/200 Mouse anti-Human TDP-43 (R&D Systems). Cells were then washed three in 0.1% (v/v) Triton X-100 in PBS and incubated with secondary antibodies and 1 μ g/mL DAPI (Sigma) for 2 h at RT. The following secondary antibodies were used in this study: anti-Mouse IgG2a AF647 (Thermo Fisher Scientific), anti-Mouse IgG2a AF488 (Thermo Fisher Scientific), anti-Rabbit AF488 (Thermo Fisher Scientific) anti-rabbit AF647 (Thermo Fisher Scientific), anti-Mouse IgM AF405 (Abcam), anti-Mouse IgG1 AF647 (Thermo Fisher Scientific), anti-Rat AF555 (Thermo Fisher Scientific). All secondary antibodies were diluted at 1/1000. Cells were final washed three time in PBS. Cells were then imaged using a Leica TCS SP8 confocal inverted laser scanning microscope at a 63× oil objective.

### RT-qPCR

Total RNA was extracted using a PureLink RNA kit (Invitrogen) according to manufacturer’s instructions, and subsequently reverse transcription was performed using a GoScript reaction kit (Promega) to produce cDNA. 5 ng of cDNA was loaded per RT-qPCR reaction using fast SYBR Green PCR Master Mix (Thermo Fisher Scientific) and reactions run on a CFX96 RT-PCR detection system (Bio-Rad). The primers are listed in the key resources table.

### Western blot

Cell pellets were generated by centrifugation at 2500 rpm for 5mins and washed once in cold PBS. Subsequently cells were lysed in NP40 lysis buffer supplemented with 1 mM PMSF, 100 mM NaV04, 1 M NaF and 1:100 protease inhibitors (Sigma-Alrich) and protein content quantified using Bradford reagent (Biorad) and a BSA standard curve (concentrations: 2, 1, 0.5, 0.25, 0.125 mg/mL) and 40 μg of protein was mixed with 4x Laemmli buffer and boiled for 10min. Samples were then loaded into a precast 10% polyacrylamide gel (Biorad) and run at 100V for 15 min then 120V until complete. Samples were then transferred using the Biorad blotting system. Samples were blocked for 1 h at RT in blocking buffer: TBST with 5% (w/v) dry milk powder and 0.1% (w/v) sodium azide, washed once with TBST and then incubated with primary antibody overnight at 4°C in blocking buffer. Antibodies to TDP-43 (Proteintech), and vinculin (Proteintech) were used at 1:1000. Samples were then washed Three times in TBST then incubated with HRP-conjugated secondary antibodies (BioRad) in blocking buffer (without sodium azide) for 1.5 h at RT. Samples were then washed three times in TBST and developed using ECL substrate (Promega) and visualised using a BioRad ChemiDoc Touch.

### Electrophysiology

For patch clamp recordings 50.000 MACS-sorted MNs and 50.000 MACS-sorted mouse ESC-GDNF astrocytes were seeded onto 1:50 Matrigel-coated 18 mm glass coverslips and cultured in MN maturation medium, comprising basal medium plus 0.1 μM RA, 0.1 μM purmorphamine and 10 μM Y-27632. Coverslips were transferred to an open bath chamber (RC-41LP Warner Instruments) containing extracellular solution: NaCl 136 mM, KCl 2.5 mM, HEPES 10 mM, MgCl_2_ 1.3 mM, CaCl_2_ 2 mM, Glucose 10 mM, pH adjusted to 7.3 and osmolarity adjusted to 300 mOsm. The chamber was mounted on an inverted epifluorescence microscope (Olympus IX71) and visualised using a 60× oil objective. Pipettes were pulled from borosilicate glass (O.D. 1.5mm, I.D. 0.86mm, Sutter instruments) to a resistance of between 3 and 5MΩ. Intracellular solution contained the following: 125 mM KMeSO_4_, 5 mM MgCl_2_, 10 mM EGTA, 10 mM HEPES, 0.5 mM NaGTP, 5 mM Na_2_ATP, pH = 7.4, osmolarity = 290mOsm. Whole-cell patch clamp recordings were then made at the soma of CHR2-YFP^+^ MNs using a Multiclamp 700B amplifier (Molecular Devices) and the data acquired using a Digidata 1440A digitizer (Molecular Devices). All recordings were carried out at room temperature. Data was acquired with Clampex software (Molecular Devices) and Axon Multiclamp Commander Software (Molecular Devices). Current-clamp data was sampled at a rate of 50 kHz and filtered at 10 kHz and voltage-clamp data was sampled at 20 kHz and filtered at 10 kHz. Whole-cell currents used to estimate Na^+^ and K^+^ conductances were recorded in voltage-clamp using 50 ms voltage steps from −80 mV to +50 mV. Resting membrane potentials and spontaneous AP spiking were recorded for 1 min in current clamp mode without current injection, within the first 2 min after break-in. Intrinsic excitability measurements and AP properties were recorded in current clamp mode, while using a steady current injection to maintain membrane potential close to −60 mV (values for injected current and holding membrane voltage were indistinguishable across genotypes). We used either 100 ms current injections from −20 pA to 170 pA (for measurements of AP properties) or 500 ms current injections from −50 pA to 300 pA (for measurements of Input-output characteristics). Although typically shorter current steps are used for establishing AP properties (e.g., - current threshold), these were unreliable at eliciting APs in younger MNs (2-week cultures). We therefore used 100 ms current steps instead, which allowed us to compare AP properties across all stages. Assessment of ’responders’ to optogenetic stimulation (Figure S2E) was carried out by delivering 500 ms pulses of 488 nm light using a CoolLED pE-100 illumination system and AP traces recorded in current clamp with membrane potential set to −60 mV.

### Microdevice fabrication and neuromuscular co-cultures

Microdevices were made according to our previously published work^28,57^ with minor modifications. A thin layer of NOA-73 resin (Norland products) was applied to plastic bottom dishes (diameter, 35 mm; ibidi, 81156) with a cell scraper and partially UV–cured for 10 s at 55 J/cm^2^. Then, the PDMS arrays were placed on the resin and fully UV-cured for 1 min. Following UV-sterilization of the devices, two neural spheroids comprising 10k MACS sorted MNs and 5k MACS sorted astrocytes formed in Lipidure coated (Amsbio) U-bottom 96 well plates were loaded into each of the two outer-compartments. Co-cultures were optogenetically entrained using 5 Hz 450 nm optogenetic stimulation for 1hr a day for 4 days, 3 days after plating 20k myoblasts in the central compartment, in order to enhance neuromuscular synapse formation.

### Optogenetic stimulation

To induce plasticity in response to short-term increases in neuronal activity we optogenetically stimulated cultures using a custom-built heat sink and LED assembly.^57^ Custom written software controlled the timing of LED emission. LEDs emitted pulses of 450nm blue light set at an LED intensity of 40%. Neurons were stimulated with bursts of 20 ms light flashes delivered at 20 Hz, with a 1 s interval between bursts, for a total of 3 h. Cultures were supplemented with 0.1% (v/v) antioxidant supplement (Sigma-Aldrich) to mitigate the effects of phototoxicity.

### Postmortem AIS staining

Frozen spinal cord was obtained from the London Neurodegenerative Disease Brain Bank. Control case 1: male 63 - Braak stage II; control case 2: male 74 - Braak stage I; ALS-1: male 55 - sporadic ALS; ALS-2 male 57 - TDP-43^M337V^ carrier; ALS-3 male 45 – TDP-43^K181E^ carrier. Tissue was sectioned on the cryostat at 20μM thickness. Sections were fixed in paraformaldehyde for 30 min and then incubated in methanol/hydrogen peroxide (0.3%) solution for 10 min to block endogenous peroxidase activity. Slides were then incubated in 10% non-fat milk for 30 min at room temperature to block non-specific binding followed by incubation with primary antibody for 1h at room temperature. The antibodies used in this study were mouse monoclonal ChAT (Choline acetyltransferase, CL3173, Novus Biologicals NBP2-46620 at 1:1000) and Ankyrin-G (N106/36, Antibodies Inc, 75–146 at 1:400). ChAT staining was carried out in order to identify MNs within the ventral horn of the spinal cords. ChAT staining was followed by three 5-min washes in tris-buffered saline with tween (TBS-T); slides were incubated for 45 min in 200 <L of biotinylated goat anti-mouse IgG secondary antibody (Vector Laboratories BA 9200, 1:200). Slides were washed as before and then incubated in pre-conjugated Strept(avidin)–Biotin Complex (ABC; DAKO) for signal amplification. The slides were then washed for a final time before being submerged in 3,3′-Diaminobenzidine (DAB) chromogen and then counterstained in Mayer’s haematoxylin (BDH). Finally, slides were dehydrated in increasing grades of alcohol (70, 90 and 100% IMS), cleared in xylene and mounted. Sections incubated with AnkG primary antibody were washed three times in PBS, followed by secondary antibody incubation (Invitrogen Goat Anti-Mouse IgG Alexa Fluor 488, 1:500) for 1 h. Sections were then rewashed and treated with 0.1% Sudan Black in 70% Ethanol for 10 min to quench any autofluorescence. Sudan black was then washed off with 30% Ethanol and sections were incubated with DAPI for 10 min (Biotium, 1:1000) followed by mounting in VectaShield (Vector Laboratories). For AIS imaging ventral spinal motor neurons were identified based on their large soma and nuclear morphology and imaged on a Zeiss LSM 980 airyscan microscope across a 20 μm z stack at 1 μm intervals.

### Analysis of AIS morphology

AIS length, diameter and start position were analyzed using FIJI. AnkG fluorescence was uniformly thresholded and the AIS length and position relative to the soma, as determined by the CHR2-YFP counter stain, were traced. A subset of AIS data (2-week) was analyzed again in a blinded manner and compared to the original measurements. Length measurements were highly consistent between the original and the blinded analyses (Corrected length: difference mean 3.904 ± 3.891, p = 0.32, r2 = 0.76. G298S length: difference mean 2.959 ± 5.299, p = 0.56, r2 = 0.76). Postmortem tissue was imaged and analyzed entirely blinded. 3D reconstructions of the AIS were carried out using IMARIS 9.1.2 software by generating a surface map based on the AnkG fluorescence. Gating thresholds for this were kept constant between samples.

### Analysis of axon outgrowth and detection of NMJs

Axon outgrowth and neuromuscular junctions were analyzed using IMARIS 9.1.2 software. For axon outgrowth, 3D surface maps were generated based on TUBB3 immunofluorescence using uniform gating thresholds. From these reconstructions the total axon volume and surface area per field of view were calculated. For analysis of neuromuscular junctions, the IMARIS coloc function was used to automatically generate a colocalization channel for the pre-synaptic SV2 immunofluorescence channel and the post-synaptic AChR channel. 3D surface maps were then generated based on this coloc channel using uniform gating thresholds. From this surface map the total number of colocalised objects as well as the volume and surface area of these objects could be calculated.

### Particle image velocimetry (PIV) analysis of myofiber contractions

Video recordings of spontaneous and optogenetically evoked myofiber contractions were analyzed by Particle Image Velocimetry using the PIVlab package^59^ in MATLAB. Three iterations of interrogation windows of 64/32/16 pixels, each with 50% overlap were used and frames calibrated to a known reference distance. Vectors were then validated by filtering out velocity values higher than 7 times the standard deviation. Mean velocity values for the myofiber compartment area were exported to derive peak velocity magnitude values and contraction frequency values. To show that contractions were dependent on synaptic transmission at NMJs, 50 μM of the AChR agonist d-Tubocurarine (DTC) (Sigma) was added to the co-cultures.

### Analysis of electrophysiological measurements

Electrophysiological measurements were analyzed with custom MATLAB scripts. Inward currents in voltage clamp were measured by taking the minimum value of a current trace, whereas steady state outward currents were measured by averaging over a 15ms window taken 25 ms after the voltage step. Values were corrected for baseline current offset before stimulation. Individual AP properties in current clamp were obtained using sequential injection of 100 ms current steps of increasing amplitude (10 pA increments). Only the first AP at the current threshold (first step to elicit an AP) was measured. AP waveforms were extracted using the MATLAB’s findpeaks function with minimum peak Amplitude 0 mV). Extracted parameters were: Amplitude (Max amplitude – average Vm at the end of the 50 ms stimulus window, excluding APs), Voltage Threshold (Voltage at the time the speed of Vm rise is above 0.15 mV/ms) and Width at half height. Input-Output parameters were obtained using sequential injection of 500 ms current steps of increasing amplitude (50 pA increments). APs were extracted using MATLAB’s findpeaks function with minimum peak Amplitude 0 mV. For analysis described in Figure S4, firing patterns were classified as: **no AP** (No AP detected at any current injection), **single AP** (maximum 1 AP detected at any stimulation intensity), **adaptive trains of APs** (multiple APs detected at at least one stimulation intensity, but frequency decrease with increasing stimulation) and **mature repetitive AP firing** (AP frequency increases monotonically with increasing stimulation strength, without frequency adaptation). Cells with a series resistance greater than 30 MΩ or a holding current lower than −100 pA were rejected.

## Quantification And Statistical Analysis

Statistical analysis was performed using Prism 9 (GraphPad) and MATLAB. AIS, electrophysiology and neuromuscular co-culture data was generally pooled from three independent experiments unless otherwise stated in the figure legend. One-Way-ANOVA with Dunnet’s test for comparisons, two-Way ANOVA, unpaired, non-parametric t-tests and Mann Whitney tests were used to infer statistically significant differences between samples and groups of samples. p-values <0.05 were deemed to be statistically significant and are denoted by *, **p < 0.01, ***p < 0.001, ****p < 0.0001. All values are represented as the mean ± SEM.

## Supplementary Material

**Supplemental Information**

Supplemental information can be found online at https://doi.org/10.1016/j.celrep.2023.113509.

Supplementary Information

Video S1

Video S2

## Data Availability

This paper does not report original code. Any additional information required to reanalyze the data reported in this paper is available from the lead contacts upon request.

## Highlights

- Increased AIS length and intrinsic hyperexcitability in ALS hiPSC motor neurons
- Impaired AIS plasticity and homeostatic regulation of excitability in ALS neurons
- Increased spontaneous myofiber contractions of *in vitro* ALS hiPSC motor units
- Progression to AIS shortening and hypoexcitability in late ALS hiPSC motor neurons
