# Supplementary material for: Aberrant axon initial segment plasticity and intrinsic excitability of ALS hiPSC motor neurons
Source: Cell Rep. Author manuscript; Available in PMC 2025 Dec 9. (PMC7618452; doi:10.1016/j.celrep.2023.113509)
Supplement: Supplementary Information [file EMS210902-supplement-Supplementary_Information.pdf]

Cell Reports, Volume *42*

Supplemental information

Aberrant axon initial segment plasticity and intrinsic excitability of ALS hiPSC motor neurons

Peter Harley, Caoimhe Kerins, Ariana Gatt, Guilherme Neves, Federica Riccio, Carolina Barcellos Machado, Aimee Cheesbrough, Lea R'Bibo, Juan Burrone, and Ivo Lieberam

Supplementary information

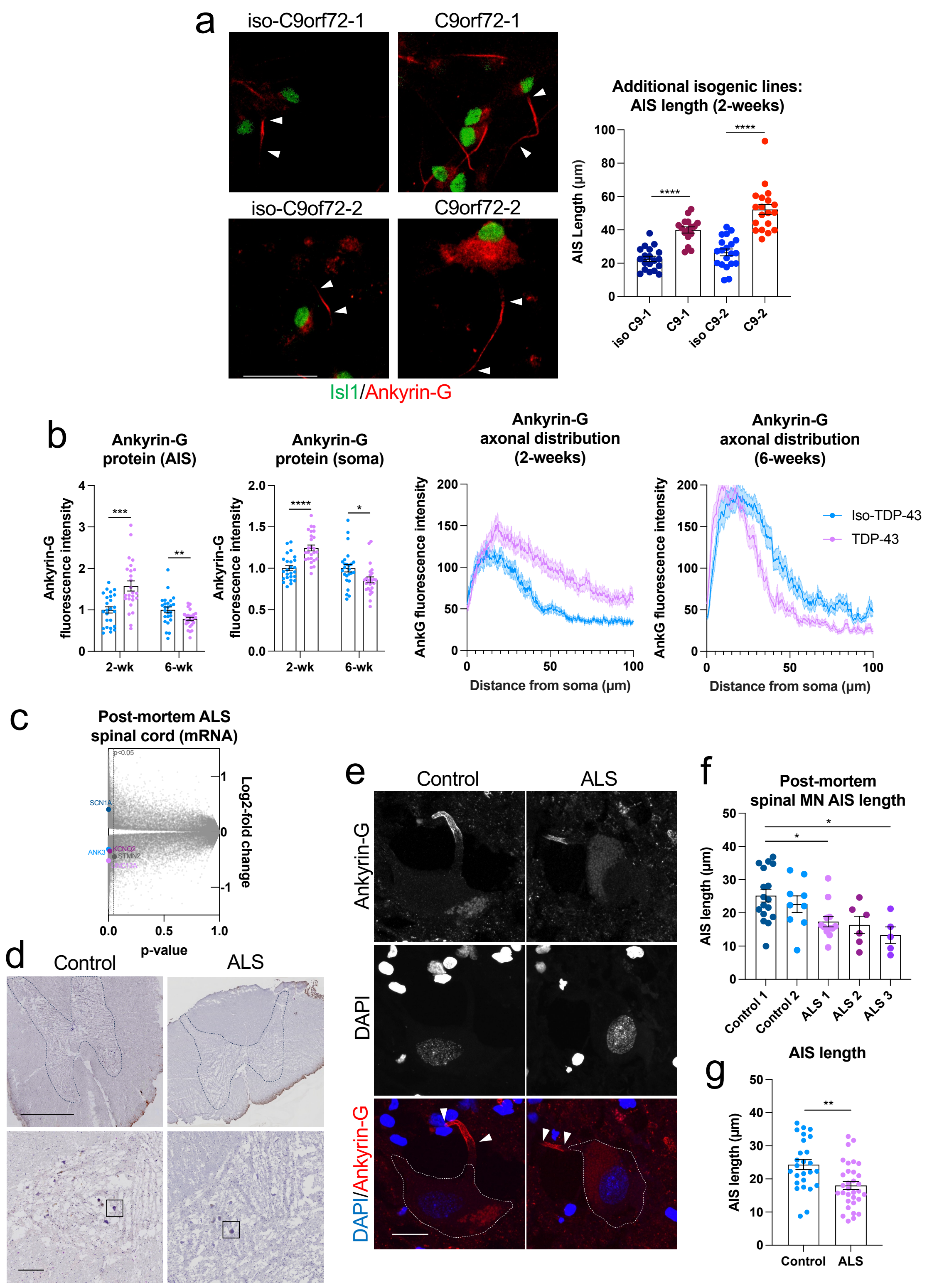

Figure S1. Additional cell lines and post-mortem data related to Figure 1 & 4. (A) AIS measurements in two additional patient *C9orf72* mutant lines and CRISPR corrected isogenic controls: C9-1 (n=19), iso-C9-1 (n=16), C9-2 (20), iso-C9-2 (n=19) at the early 2-week timepoint. Co-stained for the MN marker Isl1. Scale bar = 50µm. **(B)** Analysis of AnkG

IF protein intensities in somatic and axonal compartments at 2- and 6-weeks in iso-TDP-43^{G298S} (n=25) and TDP-43^{G298S} (n=25) MNs. Analysis of AnkG protein distribution along the axon. **(C)** Analysis of existing RNA-seq data of post-mortem pan-ALS spinal cord tissue shows mis-regulation of AIS genes: *ANK3* (****padj<0.0001), SCN1A (**padj<0.01), KCNQ2 (*padj<0.05). STMN2 and UNC13A also highlighted. **(D)** CHAT staining of control and ALS post-mortem spinal cord sections shows MN staining in the ventral horns (black square), scale bars =2mm and 200µm. **(E)** AnkG staining in ventral horn MNs in control and ALS post-mortem spinal cord, co-stained for DAPI. Scale bar = 20µm. **(F)** Analysis of AIS length based on AnkG staining in individual control and ALS cases. ALS-1 = sporadic ALS, ALS-2 = TDP-43^{M337V} carrier, ALS-3 = TDP-43^{K181E} carrier. **(G)** Comparison of AIS length from combined control and ALS cases. Error bars represent the SEM. p-values from one-way ANOVA with Tukey's multiple comparisons test (S1a,f), and unpaired t-tests (S1b,f). *p<0.05, **p<0.01, ***p<0.001. ****p<0.0001.

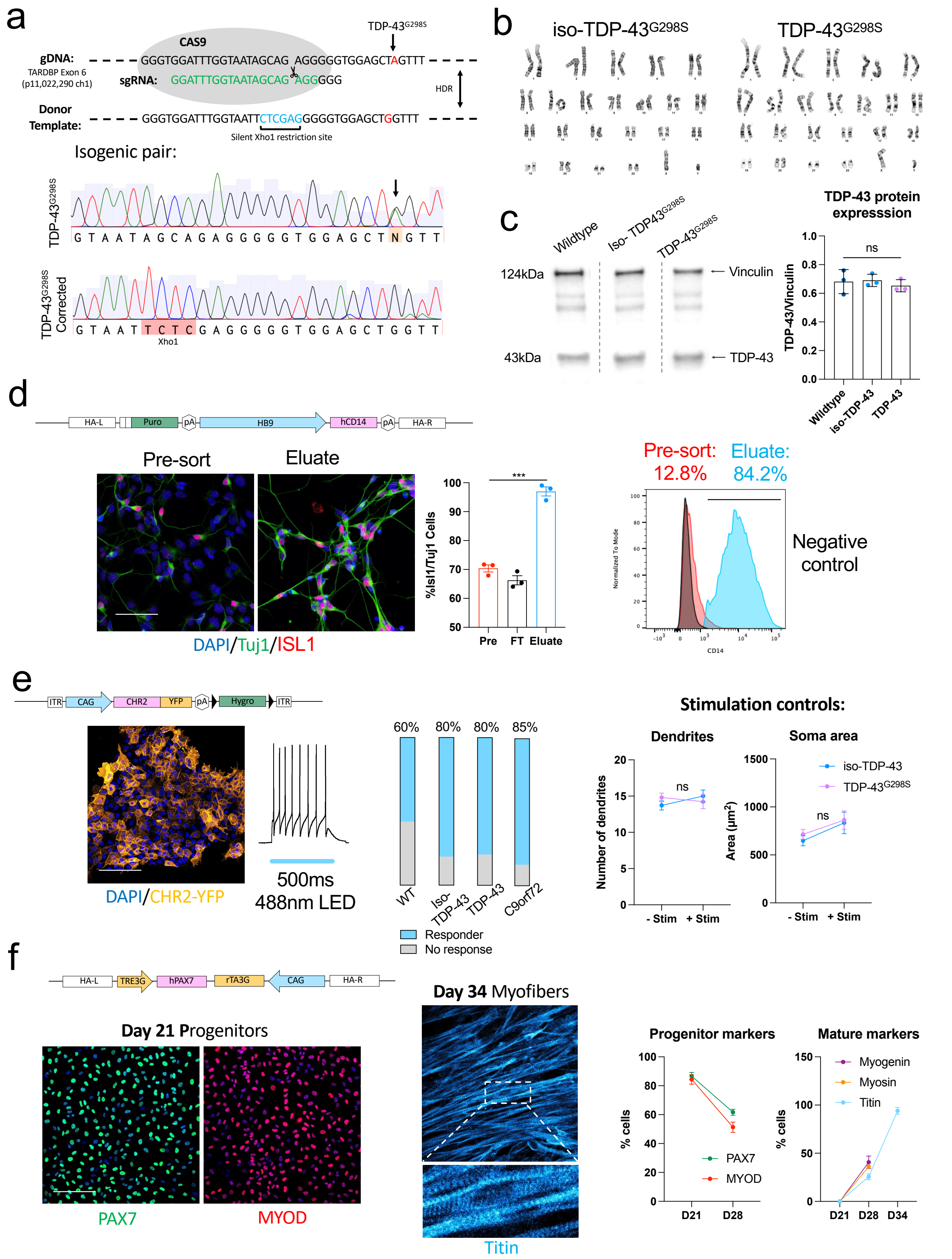

Figure S2. Generation of genetically engineered hiPSC lines related to Figure 1, 2, 3 & 4. **(A)** CRISPR-Cas9 mediated genome correction of TDP-43^{G298S} mutation. Schematic showing editing strategy and Sanger sequencing showing integration of corrected allele and silent Xho1 restriction site. **(B)** G-banding showing normal karyotype following CRISPR editing. **(C)** Western blot showing comparable protein expression of TDP-43. **(D)** hiPSC-lines engineered to stably express HB9::CD14 MACS-sortable construct. Immunofluorescence staining and

quantification for MN-specific ISL1 enrichment following MACS sorting. Flow cytometry analysis confirming MACS enrichment of HB9::CD14 MNs. Scale bar = 50µm. **(E)** hiPSC-lines engineered to stably express the optogenetic construct CAG::CHR2-YFP. hiPSC-MNs expressing CHR2-YFP reliably fire trains of action potentials in response to blue light (488nm) stimulation. Quantification of proportion of cells that fire APs in response to optogenetic stimulation. Control quantification of changes in neuronal morphology following optogenetic stimulation. Scale bar = 50µm. **(F)** hiPSC-lines engineered to stably express DOX-inducible PAX7 overexpression construct for forward programming hiPSCs into myoblasts. Immunofluorescence images of myoblast progenitors expressing PAX7 and MYOD at D21, followed by Titin expression and myoblast fusion at D34. Quantification of early and mature myogenic markers from immunofluorescence images. Scale bar = 50µm. ***$p<0.001$, error bars represent SEM.

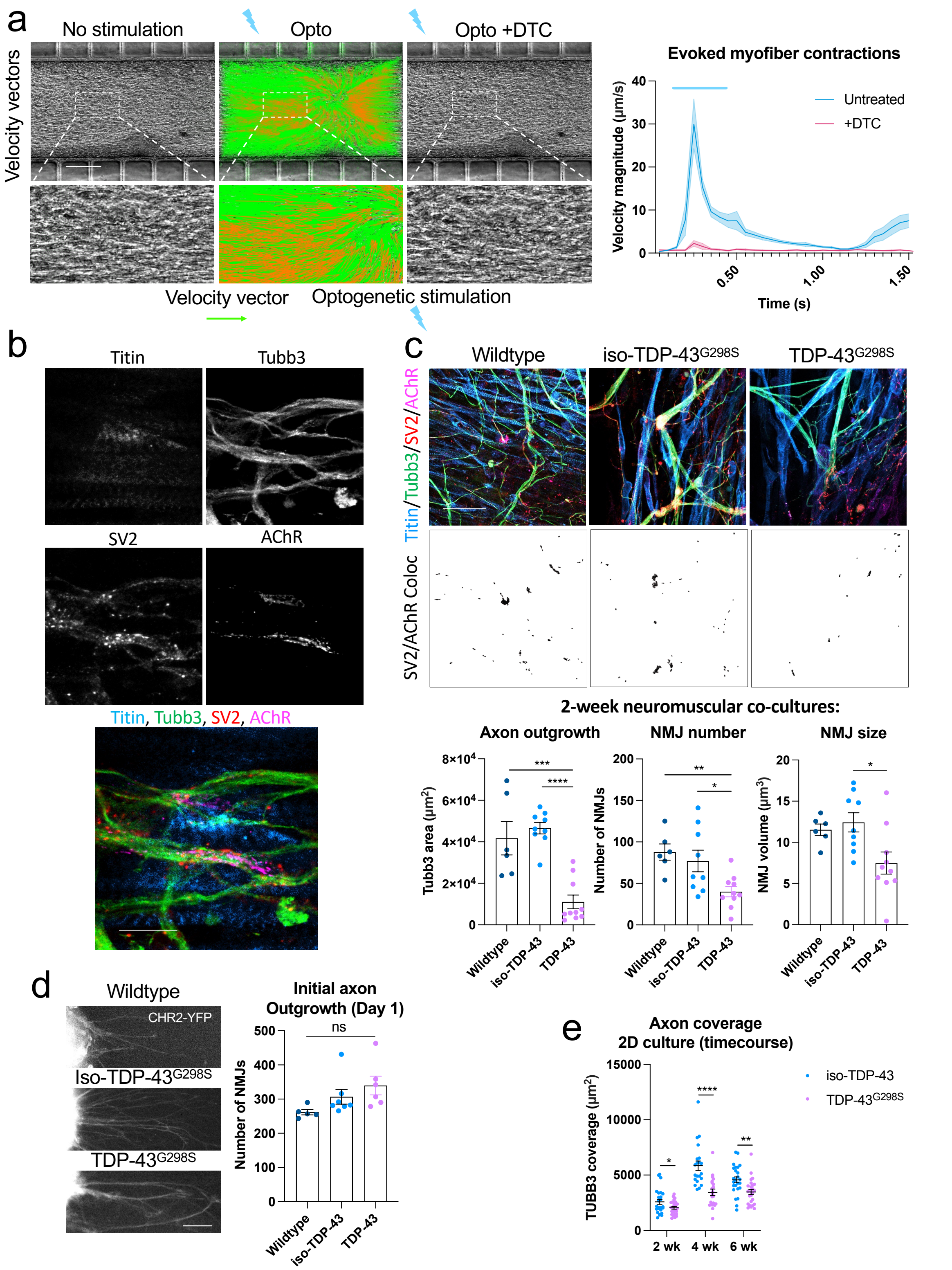

Figure S3. Additional neuromuscular co-culture data related to Figure 3. (A) PIV analysis of optogenetically evoked myofiber contractions in iso-TDP-43^{G298S} corrected neuromuscular co-cultures before and after addition of d-Tubocurarine (DTC). Scale bar = 200µm. Scale bar = 50µm. **(B)** High magnification immunofluorescence staining of neuromuscular co-cultures for Titin (cyan), Tubb3 (green), SV2 (red) and AChR (magenta). Scale bar = 50µm. **(C)** IF images of neuromuscular co-cultures pooled from 3 independent

experiments, containing wildtype (n=6), iso-TDP-43^{G298S} (n=9) and TDP-43^{G298S} (n=9) MNs. Quantification of axon outgrowth based on Tubb3 staining, and neuromuscular synapse number and size based on SV2/AChR colocalization. **(D)** Quantification of D1 axon outgrowth in the neuromuscular co-cultures based on live CHR2-YFP expression across genotypes. **(E)** Quantification of axonal coverage based on Tubb3 staining in 2D motor neuron cultures pooled from 3 independent experiments: iso-TDP-43^{G298S} 2-week (n=25), 4-week (n=21), 6 week (n=25) and and TDP-43^{G298S} 2-week (n=26), 4-week (n=21), 6 week (n=25). Error bars represent the SEM. p-values from one-way ANOVA tests with Dunnet's comparison (S3c,d) and unpaired t-tests (S3e). *p<0.05, **p<0.01, ***p<0.001, ****p<0.0001.

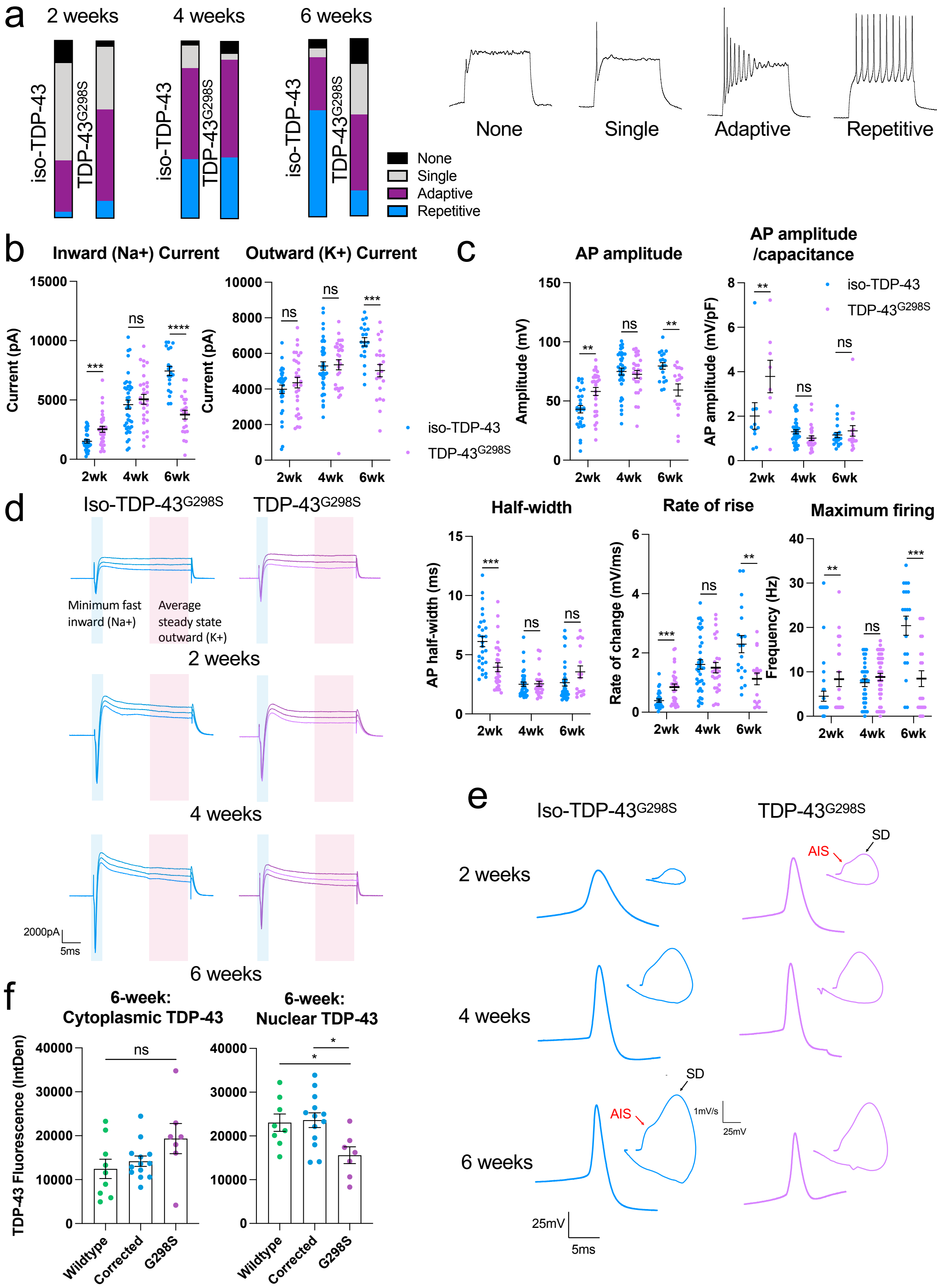

Figure S4. Changes in electrophysiological parameters overtime related to Figure 4. (A) Characterisation of firing patterns between iso-TDP-43^{G298S} and TDP-43^{G298S} lines at 2-, 4- and 6-weeks maturation from whole cell patch clamp recordings. Firing types include: no AP, single AP, adaptive trains of APs and mature repetitive AP firing. **(B)** Quantification of inward (Na+), and outward (K+) currents 2-, 4- and 6-weeks maturation, and **(C)** Quantification of AP amplitude, AP amplitude normalised to capacitance, AP half-width, AP

rate of change and maximum AP firing at 2-, 4- and 6-weeks maturation. **(D)** Representative inward (Na+) and outward (K+) current traces overtime taken at 0,10, and 20mV pulses. **(E)** Representative single AP traces and AP phase plots overtime. **(F)** Quantification of absolute nuclear and cytoplasmic TDP-43 intensities in 6-week MNs. Error bars represent the SEM. p-values from one-way ANOVA tests with Dunnet's comparison (S4f) and unpaired t-tests (S4b,c). *p<0.05, **p<0.01, ***p<0.001, ****p<0.0001.

2 weeks:	Wildtype	Wildtype +Stim	iso-TDP-43^{G298S}	iso-TDP-43^{G298S} +Stim	TDP-43^{G298S}	TDP-43^{G298S} +Stim	C9orf72	C9orf72 +Stim	One-way ANOVA
**RS**	16.28 ± 1.67MΩ	16.08 ± 1.58MΩ	16.70 ± 3.84MΩ	14.58 ± 1.54MΩ	16.94 ± 1.48MΩ	14.28 ± 2.07MΩ	9.181 ± 0.65MΩ	9.515 ± 0.56MΩ	*p=0.014
**RM**	1063 ± 141MΩ	1140 ± 176MΩ	714 ± 69MΩ	1207 ± 314MΩ	786 ± 106MΩ	911 ± 176MΩ	753.1 ± 166.3MΩ	594 ± 77MΩ	p=0.055
**CM**	20.67 ± 2.49pF	21.21 ± 2.73pF	23.49 ± 2.02pF	25.96 ± 2.71pF	19.79 ± 3.51pF	30.10 ±3.60pF	31.63 ± 2.86pF	33.76 ± 2.60pF	**p=0.007
**Resting voltage**	-35.95 ± 2.85mV	-31.52 ± 2.45mV	-38.71 ± 1.20mV	-35.78 ± 2.25mV	-38.91 ± 2.84mV	-34.09 ± 2.90mV	-47.66 ± 3.11mV	43.57 ± 2.53mV	**p=0.007
**Current Thresh**	71.43 ± 10.47pA	90.67 ± 17.31pA	65.36 ± 6.14pA	94.00 ± 10.60pA	44.29 ± 3.20pA	65.56 ± 9.26pA	28.57 ± 2.54pA	31.67 ± 3.66pA	**** p=<0.0001
**Voltage Thresh**	-25.07 ± 2.12mV	-21.35 ± 2.74mV	-25.11 ± 1.32mV	23.47 ± 1.98mV	-26.55 ± 1.40mV	29.40 ± 1.69mV	-30.07 ± 1.38mV	-29.65 ± 1.11mV	*p=0.018
**Holding current**	-33.68 ± 4.78pA	-44.74 ± 7.62pA	-32.14 ± 5.08pA	-37.71 ± 5.59pA	-29.03 ± 6.81pA	36.67 ± 6.29pA	-21.06 ± 7.35pA	-29.07 ± 1.82pA	P=0.32
**Holding voltage**	-59.77 ± 0.76mV	-57.06 ± 1.821mV	-60.43 ± 0.91mV	-60.27± 1.11mV	-60.69 ± 0.79mV	-60.90 ± 0.52mV	-59.16 ± 0.99mV	-56.47 ± 1.45mV	P=0.051

4 weeks:	iso-TDP-43^{G298S}	TDP-43^{G298S}	Unpaired T-test
**RS**	10.54 ± 0.47MΩ	9.80 ± 0.42MΩ	P=0.27
**RM**	497 ± 42MΩ	357 ± 28MΩ	*P=0.013
**CM**	70.78 ± 5.73pF	84.8 ± 6.10pF	P=0.10
**Resting voltage**	-51.62 ± 1.58mV	-43.75 ± 2.91mV	*P=0.01
**Current Threshold**	77.57 ± 5.96pA	89.23 ± 7.19pA	P=0.22
**Voltage Threshold**	-34.9 ± 1.40mV	-32.65 ± 1.37mV	P=0.27
**Holding current**	-31.41 ± 4.39pA	-51.07 ± 6.16pA	**P=0.0094
**Holding voltage**	-60.77 ± 0.34mV	-60.42 ± 0.39mV	P=0.51

6 weeks:	iso-TDP-43^{G298S}	TDP-43^{G298S}	Unpaired T-test
**RS**	9.06 ± 0.40MΩ	11.57 ± 0.78MΩ	**p=0.007
**RM**	320 ± 36MΩ	427 ± 72MΩ	p=0.20
**CM**	88.67 ± 7.40pF	53.11 ± 6.43pF	**p=0.001
**Resting voltage**	-53.66 ± 2.30mV	-43.44 ± 3.96mV	**p=0.002
**Current Threshold**	100.00 ± 9.67pA	74.71 ± 9.59pA	p=0.073
**Voltage Threshold**	-31.95 ± 1.11mV	29.11 ± 2.3mV	p=0.248
**Holding current**	-26.25 ± 7.36pA	-47.62 ± 9.28pA	p=0.08
**Holding voltage**	-60.29 ± 1.17mV	-60.55 ± 1.10mV	p=0.87

Table S1. Passive membrane properties and other electrophysiological parameters related to Figure 1, 2 & 4. Access resistance (RS), membrane resistance (RM), capacitance (CM), resting voltage, current threshold, voltage threshold, baseline voltage, holding current and holding voltage in wildtype, iso-TDP-43^{G298S}, TDP-43^{G298S} and C9orf72-3 MNs at 2-, 4- and 6-weeks maturation with and without optogenetic stimulation. P-values from

unpaired nonparametric t-tests and one-way ANOVA tests. *p<0.05, **p<0.01, ***p<0.001, ****p<0.0001.
